# Lung Cancer Cell-intrinsic Asparagine Synthetase Potentiates Anti-Tumor Immunity via Modulating Immunogenicity and Facilitating Immune Remodeling in Metastatic Tumor-draining Lymph Nodes

**DOI:** 10.7150/ijbs.114791

**Published:** 2025-10-10

**Authors:** Ziyu Zhang, Nannan Du, Gege He, Mengting Zhang, Gaifeng Zhang, Jie Gao, Ying Liu, Yuezhen Deng, Lunquan Sun, Min Li

**Affiliations:** 1 Department of Respiratory Medicine, National Key Clinical Specialty, Branch of National Clinical Research Center for Respiratory Disease, Xiangya Hospital, Central South University, Chang-sha, China.; 2 Shanghai Chest Hospital, Shanghai Jiao Tong University School of Medicine, Shanghai, China.; 3 Department of Pathology, Xiangya Hospital, Central South University, Changsha, China.; 4 Department of Oncology, Xiangya Cancer Center, Xiangya Hospital, Central South University, Hunan Province, Changsha, China.; 5 Key Laboratory of Molecular Radiation Oncology Hunan Province, Hunan Province, Chang-sha, China.; 6 Hunan International Science and Technology Collaboration Base of Precision Medicine for Cancer, Hunan Province, Changsha, China.; 7 Xiangya Lung Cancer Center, Xiangya Hospital, Central South University, Changsha, China; 8 Clinical Research Center for Respiratory Diseases in Hunan Province, Changsha, China.; 9 National Clinical Research Center for Geriatric Disorders, Changsha, China.

**Keywords:** ASNS, lymph node metastasis, stem-like T cells, neoadjuvant immunotherapy, NSCLC

## Abstract

**Background**: In non-small cell lung cancer (NSCLC), lymph node (LN) metastasis is a crucial prognostic factor. Asparagine synthetase (ASNS) plays a crucial role in cellular aspartate metabolism and promotes LN metastasis. However, the mechanisms by which LN metastasis affects immune microenvironment remodeling *in situ* and tumor-draining LNs (TdLNs), as well as the role of ASNS in this process remains unclear.

**Methods**: LN metastatic lung cancer cell lines were established through *in vivo* selection in a murine model and subsequently analyzed via metabolomic profiling. ASNS expression and its role in modulating immunogenicity were assessed using transcriptomic analysis, western blotting, and immunohistochemistry. Metabolomic profiling, combined with *in vitro* stimulation assays, identified key metabolic regulators involved in the axis. Furthermore, T-cell kinetics were monitored via flow cytometry, multiplex immunofluorescence and patient datasets. Tissue samples from NSCLC patients with LN metastases following neoadjuvant immunotherapy were employed to validate findings.

**Results**: Elevated aspartate metabolism and ASNS expression were observed in LN metastasis based on metabolomic analyses of LN metastatic lung cancer cell lines and immunohistochemistry of tissue samples from LN metastasis, intrapulmonary implantation, LN injection models and NSCLC patients-derived samples. Higher ASNS expression in LN metastases correlated with enhanced immunogenicity. Mechanically, ASNS promoted the expression of major histocompatibility complex through α-aminobutyric acid auto-secretion in lung cancer cells. Moreover, *in vivo* and clinical studies revealed that metastatic tumor areas with high ASNS expression facilitated the formation of lymphocyte niches conducive to CD8+T cell activation, memory, and stemness within metastatic TdLNs, particularly in the vicinity of metastatic foci, thus reshaping the immune landscape in both tumors *in situ* and metastatic LNs. Clinical research confirmed that high ASNS expression in LN metastases correlated with improved efficacy of neoadjuvant immunotherapy in NSCLC patients.

**Conclusions**: ASNS promotes anti-tumor immunity in NSCLC via regulating immunogenicity of cancer cells and immune microenvironment remodeling in metastatic TdLNs. Lung cancer cell-intrinsic ASNS appears to be a promising marker for anti-PD-1-based neoadjuvant immunotherapy.

## Introduction

Non-small cell lung cancer (NSCLC) remains one of the primary causes of cancer-related mortality 1, with lymph node (LN) metastasis serving as a crucial prognostic marker in NSCLC patients 2-3. Recent studies have identified the tumor-derived lymph nodes (TdLNs) as the key site for immune modulation, where initial antigen presentation occurs, followed by T-cell activation and expansion. This process ultimately leads to the migration of primed T cells to the primary tumor site, facilitating tumor eradication and the establishment of immune memory 4-6. Furthermore, our previous study on early-stage lung cancer has also demonstrated that LN metastasis enhances tumor-specific antigen presentation in TdLNs, triggering T-cell activation and memory, and subsequently altering the immune microenvironment of primary tumors. These findings have underscored the critical role of LN metastasis in remodeling anti-tumor immunity 7. However, the immune characteristics associated with LN metastasis remain poorly understood, and whether the remodeled immune environment in metastatic TdLNs impacts immune responses at the primary tumor site has yet to be addressed.

Anti-PD-1/PD-L1-based immunotherapy has demonstrated durable remissions by reversing CD8+ T cell exhaustion 8. However, several studies showed that chronic exposure to abundant tumor antigens within the tumor microenvironment (TME) drives most CD8⁺ T cells into a state of terminal exhaustion, marked by diminished proliferative capacity and impaired effector function sustained by irreversible epigenetic reprogramming 9. Subsequent researches discovered that TCF-1-expressing, stem-like T cells (Tsl) are the primary responder of therapeutic responses to PD-1/PD-L1 blockade. These cells induce anti-tumor immunity by maintaining a pool of proliferative progenitors capable of differentiating into effector T cells, and the process can be further enhanced by ICB treatment 10-13. Besides, recent studies have further elucidated that TdLNs, rather than TME, serve as a reservoir for Tsl cells, supporting the sustained presence of antitumor CD8+ T cell and shielding them from terminal differentiation during tumorigenesis 14-15. Moreover, the Tsl subset in TdLNs has been characterized as TdLN-derived stem-like and memory T cells (CD44+CD62L+TCF1+Tox-, TTSM), which have been shown to act as primary responders to immune checkpoint blockade (ICB) treatment in preclinical models and lung cancer patients 16-18. However, all conclusions to date have been based on studies involving mice or patients without LN metastasis. Therefore, it remains unclear whether LN metastasis influences the proportion, function, and role of TTSM in PD-1/PD-L1 blockade treatment.

Asparagine synthase (ASNS) was an indispensable enzyme to catalyze the production of asparagine and glutamate from aspartate and glutamine through an ATP-dependent amido-transferase reaction, concomitant with the deamidation of glutamine 19. We previously showed that ASNS promotes cellular invasiveness of lung cancer cells through the WNT pathway and mitochondrial functions 20. Moreover, asparagine, the main product of ASNS, played a critical role in the direct modulation of the adaptive immune response by regulating T-cell activation and memory 21-22. Besides, following the ligation of TCR, asparagine regulated modulates several key steps in the signaling cascades downstream of the TCR, including boosting proximal antigen-specific signals, promoting LCK activity, and sequentially augmenting the attachment to target cells 23. Consequently, to further investigate the mechanism underlying LN metastasis on immune remodeling, we hypothesize that ASNS may regulate the immune-related characteristics of tumor cells within LN metastases, thereby modulating anti-tumor immunity.

In this study, we have identified that lung cancer cells with high expression of ASNS have an increased propensity for LN metastasis. ASNS promotes alpha-aminobutyric acid secretion, thereby augmenting the expression of MHC and antigen-presentation genes in lung cancer cells, and consequently enhancing the cytotoxicity of CD8+T cells. Furthermore, metastatic tumor regions with high ASNS expression promote the development of lymphocyte niches conducive to T cell activation, memory, and TSL and TTSM production in metastatic LNs, particularly in the vicinity of metastatic foci, which contributed to remodeling the immune microenvironment in both the tumor *in situ* and metastatic LNs, and ultimately improving the efficacy of neoadjuvant immunotherapy for patients with LN metastases.

## Materials and Methods

### Human samples

Adult patients who were histologically confirmed NSCLC with LN metastases, adequate organ function, and ECOG PS of 0-1, who had no comorbidity or required immunotherapy, were recruited. A total of 25 NSCLC patients who underwent radical surgical treatment at Xiangya Hospital were selected. Some collected human samples were stored in liquid nitrogen. Other samples were embedded in paraffin, and fixed on glass slides using formalin for IHC experiments.

Adult patients who were histologically confirmed NSCLC at stages IIa to IIIb, with LN metastases, adequate organ function, and ECOG PS of 0-1, who had no comorbidity or required immunotherapy, were recruited. A total of 18 NSCLC patients who underwent neoadjuvant anti-PD-1-based immunotherapy followed by radical surgical treatment at Xiangya Hospital between 2022 and 2023 were selected. All samples were embedded in paraffin, and fixed on glass slides using formalin for IHC experiments.

Patients were recruited in accordance with an approved Institutional Review Board (IRB) protocol, and all provided informed consent.

### Cell lines and culture

The human-derived lung cancer cell lines A549 and H1299, as well as the mouse-derived Lewis lung carcinoma (LLC) cells, were obtained from the National Collection of Authenticated Cell Cultures. A549 and H1299 cells were cultured in RPMI 1640 medium added with 10% fetal bovine serum (FBS) and 1% penicillin-streptomycin-amphotericin B (Solarbio, P7630). LLC cells were cultured in DMEM added with 10% FBS and 1% penicillin-streptomycin-amphotericin B (Solarbio, P7630).

### Plasmid

The coding sequence (CDS) of ASNS^WT^ and ASNS^C2A^ was cloned into the p23 vector. The shRNA targeting ASNS were designed using the Sigma-Aldrich online tool and subsequently inserted into the pLKO.1-puro vector. The corresponding shRNA sequences are listed in Supplementary Table 1.

### Mice

C57BL/6J (B6) mice and OTI transgenic mice, which expressed the T-cell receptor (TCR) specific for the OVA257-264 peptide in the context of H-2Kb, were obtained from Shanghai Model Organism. All mice were on the C57BL/6J background and treated at 8-10 weeks of age. Mice were raised under specific pathogen-free (SPF) conditions. All breeding and experimental procedures adhered to the Institutional Animal Care and Use Committee (IACUC) guidelines.

### Mouse Studies

All mice were provided with standard laboratory chow (PMI Lab Diet) and water. Animals were randomly divided into three groups (the control group, the ASNS^WT^ group and ASNS^C2A^ group; the control group, the shASNS-4 group and the shASNS-5 group), using a computer-based random order generator. Before all surgical and sampling procedures, mice were intraperitoneally injected with a ketamine (80 mg/kg) and xylazine (12 mg/kg) mixture to anesthetize. For the LLC footpad implantation model, 5×10⁵ LLC cells were subcutaneously injected into the footpad of the hind limb in 8- to 10-week-old C57BL/6J mice. In the intrapulmonary implantation model, 8- to 10-week-old C57BL/6J mice were intrapulmonary injected with 5 × 10⁶ cells under anesthesia, and euthanized 36 days post-injection. For LN injections, the lymphatic vessels were visualized through injecting 2% Evans blue dye (E2129, Sigma-Aldrich) into the footpad five minutes before the procedure. Following dye administration, a 5-10 mm incision was made over the popliteal LN. The LN was stabilized with forceps, and injected with 10 μL of PBS-suspended cells. The successful injection was confirmed by observable LN swelling. Euthanasia was carried out immediately after injection of 6-8W or when the mice experienced a weight loss of more than 10%, inability to feed or drink, etc. The individual mouse was considered the experimental unit within the studies, and n refers to a number of animals. In order to meet statistical requirements and minimize the use of experimental animals, the number of mice in each group was 6-8. Mice with tumors that were too large or too small, exhibiting a premature inability to feed or drink water were excluded.

### Tumor Line Generation

For LN metastatic lung cancer lines generation, mice were implanted with the syngeneic lung cancer cell line LLC, leading to the development of *de novo* LN metastases. Popliteal and inguinal LNs were harvested, mechanically dissociated, and processed with digestion using Collagenase P (Roche, 11213865001) and Dnase (Sigma, DN25-10MG) in DMEM at 37°C for 30 minutes to generate LN lines. Then LN lines were expanded *ex vivo* into cell lines and implanted into naive recipients.

### Q-PCR

Total RNA was extracted using TRIzol (Invitrogen), and 1 μg RNA was reverse-transcribed into cDNA with the PrimeScript RT kit (Takara). Quantitative PCR was performed using SYBR Green chemistry (BioRad) on a CFX96 real-time PCR system (BioRad), undergoing 4 steps (amplification steps: 95°C for 10s, 55°C for 15s, 68°C for 30s, repeating 40 circle). Gene expression was quantified using the comparative Ct (2^-ΔΔCt) method. Primer sequences are listed in Supplemental Table 2.

### Western blotting

The prepared cells were resuspended in 100-200 μL of RIPA buffer (freshly added Proteinase Inhibitor) on ice. The cell mixture was pipetted until fully dissolved and set on ice for 30 minutes. The lysis mixture was centrifuged at 12,000 rpm for 15 minutes to harvest the supernatant. Then the samples were supplemented with 5× sample loading buffer, with 200 mM DTT freshly added. The samples were loaded onto the gel for electrophoresis. Following this step, the sample underwent a constant current wet transfer at 300 mA in the cold chamber. The membranes were soaked with skim milk (diluted in PBS, 5%) for 1 hour and then incubated with the appropriate primary antibodies overnight in the cold chamber. Afterward, membranes were washed in PBS (3×3 minutes) and then incubated with the secondary antibody (diluted in skim milk, 1:3000) at room temperature for 1-2 hours. Following additional washing with PBS (3×3 minutes), protein detection was performed using a Chemiluminescence Gel Imaging System (ChampChemi 910). The antibodies used were anti-ASNS (Proteintech, 14681-1-AP), anti-HLA-ABC (Proteintech, 15240-1-AP), anti-GAPDH (Bioworld, AP0063), and anti-HSP90 (ZEN-BIOSCIENCE, 209902).

### Metabolomics analysis

The prepared samples were immediately frozen in liquid nitrogen until sent to Metabo-Profile (Shenzhen, China). The transferred samples were prepared and analyzed by the two modes (for detection of both cationic and anionic metabolites) of capillary electrophoresis time-of-flight mass spectrometry, and liquid chromatography time-of-flight mass spectrometry. Identification of metabolites from the peaks was based on the annotated tables of m/z values and normalized migration times. Relative peak areas under the curves were quantified. As the assay employed targeted metabolomics, it did not follow a universally standardized protocol. The specify analytical pipelines for differential metabolites in metabolomics are: p < 0.05, log2|fc| >= 0, and VIP > 1.

### Transcriptomics analysis

The prepared samples were added with Trizol and immediately frozen in liquid nitrogen until sent to Novogene (Wuhan, China). Gene expression levels were normalized using the fragments per kilobase of transcript per million mapped reads (FPKM) method. The specify analytical pipelines for differential expression in genes are: p < 0.05, log2|fc| >= 0, and VIP > 1.

### Tissue processing and flow cytometry

Tumors and TdLNs were stored in PBS (1% BSA). Tumor tissue was processed with digestion using Collagenase P and DNase in DMEM at 37 °C for 60 minutes prior to mechanical dissociation. LNs were processed with digestion using Collagenase P and DNase at 37 °C for 30 minutes before mechanical cutting. Cells were then stained for viability using the Fixable Viability Stain 700 (BD Pharmingen, 564997) at 4 °C for 15 minutes. Afterward, cells were washed in PBS and then were performed with surface staining at 4 °C for 30 minutes. Finally following additional washing with PBS, cells were fixed and permeabilized using the Transcription Factor Staining Buffer Set (BD Pharmingen, 562574) to enable nuclear factor staining. Intranuclear antibodies were incubated for 60 minutes at 4 °C. Antibodies used included CD3e (BD Pharmingen, 564379), CD45 (BD Pharmingen, 560510), CD8a (BD Pharmingen, 551162), CD44 (BD Pharmingen, 563114), CD62L/Sell (BD Pharmingen, 560516), TCF1/TCF7 (BD Pharmingen, 566692), and Tox (Thermo, 50-6502-82).

### OT-I Co-Culture Studies

OT-I mice were housed at Central South University. Spleens were obtained from OT-I mice and then mechanically dissociated and washed with PBS (1% BSA). Red blood cells were destroyed with the Ammonium-Chloride-Potassium (ACK) lysing buffer. CD8+T cells were harvested using the EasySep™ Mouse CD8+T Cell Isolation Kit (StemCell Technologies, 19853). A total of 3×10^5^ CD8+T cells were co-cultured with 1.5×10^5^ tumor cells (LLC-Ova-EV, LLC-Ova-ASNS^OE^, LLC-Ova-ASNS^C2A^) in 100 µL of RPMI-1640 medium added with 10% FBS, 15 mM HEPES, 2 mM L-glutamine, and 14.3 mM 2-mercaptoethanol. After being co-cultured for 24-48 hours, cells were obtained for flow cytometry analysis. Staining and analysis were described in the "Tissue Processing and Flow Cytometry" section.

### IHC and Fluorescent multiplex IHC (mIHC)

Place FFPE tissue slides in a 65 °C oven to melt the paraffin surrounding the tissue. The slides were deparaffinized using xylene, hydrated with graded ethanol solutions, rinsed with running water for 3 minutes, and boiled in EDTA buffer (Sigma) at pH 8 for 20 minutes at 95 °C. Then the FFPE tissue slides were incubated in endogenous peroxidase inhibitor for 30 minutes. After washing (3×5 minutes with PBS), the slides were then reacted with 5% BSA in PBS for 30 to 60 minutes. The slides were incubated with primary antibody at 20-25 °C for 30-60 minutes or 4 °C overnight, followed by washing (3×5 minutes with PBS), then reacted with secondary antibody at 20-25 °C for 10 minutes. Subsequently, incubate the slide with DAB for IHC while incubate the slide with 1x TSA colorimetric working solution for mIHC at room temperature for 5 minutes, followed by three washes with PBS. Nuclei were stained with hematoxylin for IHC and DAPI for mIHC. Multi-channel tissue staining images were scanned and captured by the Vectra 3.0 imaging system with a multispectral microscope. InForm 2.4.0 image analysis software (Akoya Biosciences and PerkinElmer) was used for the phenotypic recognition and quantitative analysis of T cell subtypes. For mIHC, the mouse tissue slides were sequentially labeled with monoclonal antibodies against CD44 (Bosterbio, A00052), CD62L (Bosterbio, PB9389), CD8 (Bosterbio, A02236-1), Ovabumin (Abcam, ab181688), and TCF-1 (Cell Signaling Technology, 2203T). Similarly, the human tissue slides were labeled with monoclonal antibodies against CD45RO (ZSGB-Bio, ZM-0055), CD62L (Bosterbio, PB9389), CD8 (Proteintech, 66868-1-lg), CK (ZSGB-Bio, ZM-0069), and TCF-1 (Cell Signaling Technology, 2203T). All markers were stained sequentially using their corresponding fluorophores from the Opal 7 kit (lot 20203002; Akoya Biosciences and PerkinElmer, Waltham, MA).

### Transwell

70 ul of cytokine-free Matrigel, diluted at a ratio of 30:1000 in basal DMEM, was applied to the center of the bottom surface of the upper chamber membrane. After 30 minutes of incubation, the supernatant was carefully removed. A total of 20,000 cells were seeded in the upper chambers of Transwell plates (Corning, 3422) containing DMEM supplemented with 0.5% FBS, while the lower chambers were filled with DMEM containing 2% FBS to establish a chemotactic gradient. Following 24 hours of incubation at 37 °C in a humidified 5% CO₂ atmosphere, migrated cells were fixed with 4% paraformaldehyde, stained with crystal violet, and quantified by counting nine random microscopic fields per insert.

### 68Ga-Pentixafor PET/CT Scans and Image Analysis

Gallium-68 was eluted from a 68Ge/68Ga generator (GalliaPharm; Eckert & Ziegler Radiopharma GmbH), and radiolabeling of pentixafor with 68Ga was performed in accordance with the manufacturer's protocol. Imaging studies were conducted using a dedicated PET/CT scanner (Discovery 690 Elite; GE Healthcare). Patients underwent imaging under standard dietary conditions without specific preparation. 68Ga-pentixafor was administered intravenously at a dose of 148-185 MBq (4-5 mCi). Approximately 30 minutes post-injection, low-dose CT of the adrenal region was acquired, followed by an 8-minute PET acquisition using a single bed position. PET images were reconstructed with a three-dimensional ordered-subsets expectation maximization (OSEM) algorithm using 2 iterations and 23 subsets.PET/CT images were independently evaluated by two board-certified nuclear medicine physicians who were blinded to all clinical information. The maximum standardized uptake value (SUVmax) of each pulmonary lesion was measured across the lesion area, using a threshold of 2.5 to distinguish between benign and malignant lesions.

### Statical analysis

Statistical analysis was performed by utilizing SPSS version 23 (IBM Corporation) and GraphPad Prism version 8.0 (GraphPad Software). Kaplan-Meier survival analysis, as well as the Log-rank (Mantel-Cox) test, was employed to evaluate patient survival outcomes. Between-group variations were tested using unpaired t-tests, paired t-tests, or ANOVA. P-value < 0.05 was considered statistically significant.

## Results

### Aspartate metabolism was elevated in LN metastatic tumor lines

**Figure 1:**
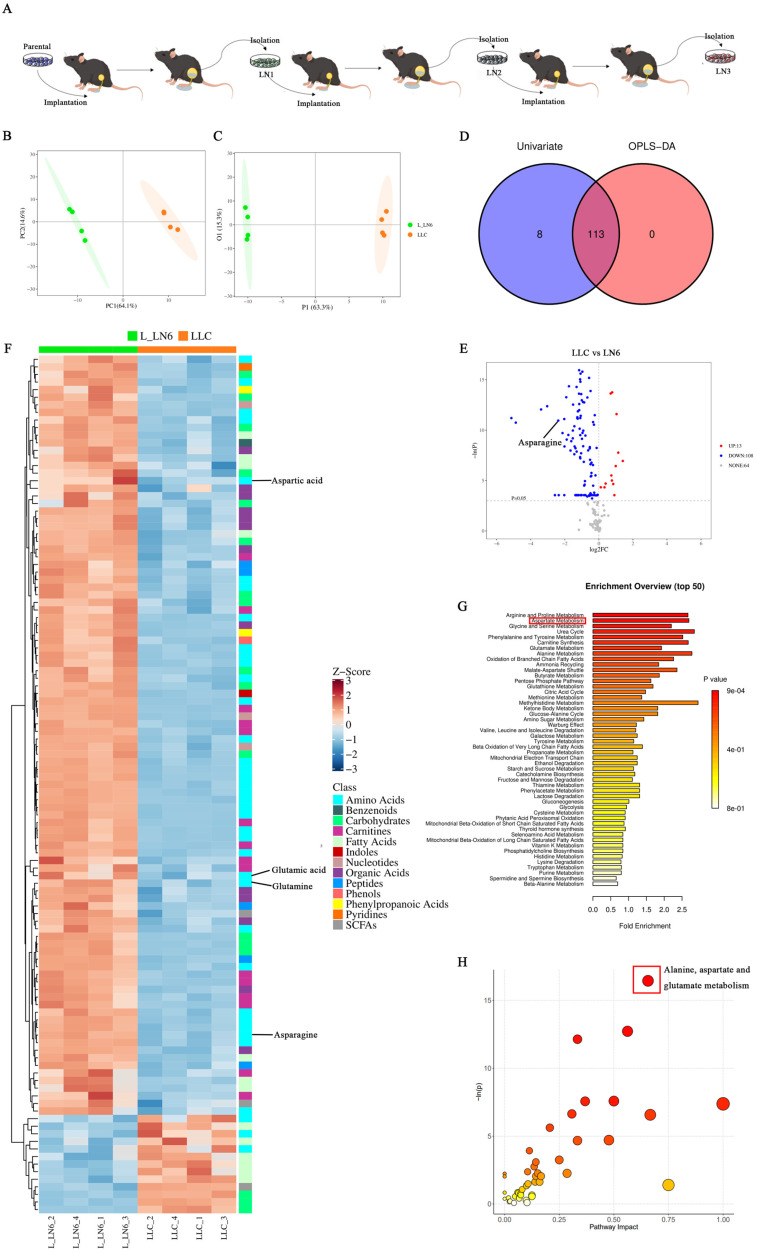
** Aspartate metabolism was elevated in LN metastatic tumor lines. Figure 1A**. LN metastatic tumor lines were generated through serial *in vivo* selection of LN metastases. **1B-C**. PCA 2D score plot (B) and OPLS-DA 2D score plot (C) of LLC and L-LN6. **1D**. Venn Plot of differential metabolites from multi-dimensional statistics and univariate statistics. **1E**. Volcano plot depicting metabolite differences based on univariate statistical analysis, in which differential metabolites (points with red highlight) in the right top corner are increased in LLC and differential metabolites (points with blue highlight) in the left top corner are decreased in LLC compared with L_LN6. **1F**. Heatmap of significantly altered metabolites. **1G-H**. Pathway enrichment analysis using pathway-associated metabolite sets (SMPDB) (G) and pathway analysis bubble plot (H) reveal upregulated and downregulated metabolic pathways in L-LN6 relative to LLC.

To explore the impact of LN metastasis on immune microenvironment remodeling in lung cancer and LNs, we attempted to characterize the tumor cells within LN metastases. To establish LN metastatic tumor lines, we adopted an *in vivo* selection strategy analogous to those used in organotropic metastasis models 24-27. Mice were implanted with the syngeneic lung cancer cell line LLC, leading to the development of *de novo* LN metastases. LN-metastases-derived cell lines were subsequently generated and implanted into naive recipients. (**Figure 1A**). This process was repeated over six generations. Metabolomic analysis was then performed on both the parental LLC cells and the sixth-generation LN-metastases-derived cells (L-LN6). Principal component analysis (PCA) and orthogonal partial least squares discriminant analysis (OPLS-DA) demonstrated distinct clustering of parental LLC cells and L-LN6 cells, indicating significant metabolic divergence (**Figure 1B-C**). By intersecting differential metabolites identified through univariate and multi-dimensional statistical analyses, we identified a total of 113 significantly altered metabolites in amino acids, nucleotides, organic acids, carbohydrates, SCFA, fatty acids and other metabolite classes (**Figure 1D**). Volcano plots using univariate statistical analysis identified 13 upregulated and 100 downregulated differential metabolites in the parental LLC group (**Figure 1E**). Notably, metabolites associated with aspartate and asparagine metabolism were enriched after *in vivo* selection (**Figure 1F**). To identify the biological changes in L-LN6 cells, we performed pathway-associated metabolite set analysis (SMPDB) to classify differential metabolites and highlighted the top 50 terms (**Figure 1G**). Statistical analysis revealed a significant enrichment of differential metabolites in the aspartate metabolism pathway. Moreover, pathway analysis using mmu set also indicated an upregulation of aspartate metabolism in LN metastatic lung cancer lines (**Figure 1H**). It has been proven that ASNS is a crucial enzyme involved in cellular aspartate metabolism 19. We previously demonstrated that elevated ASNS expression in primary tumors correlated with a higher incidence of lymph node metastasis and enhanced the invasive capacity of lung cancer cells 20 (**Supplemental Figure 1**). Therefore, we designated ASNS as a focal point to investigate the mechanisms through which LN metastasis modulated immune microenvironment remodeling in both the primary tumor site and TdLNs.

### Tumor cells with ASNS high expression were predisposed to metastasis in lymph nodes

**Figure 2:**
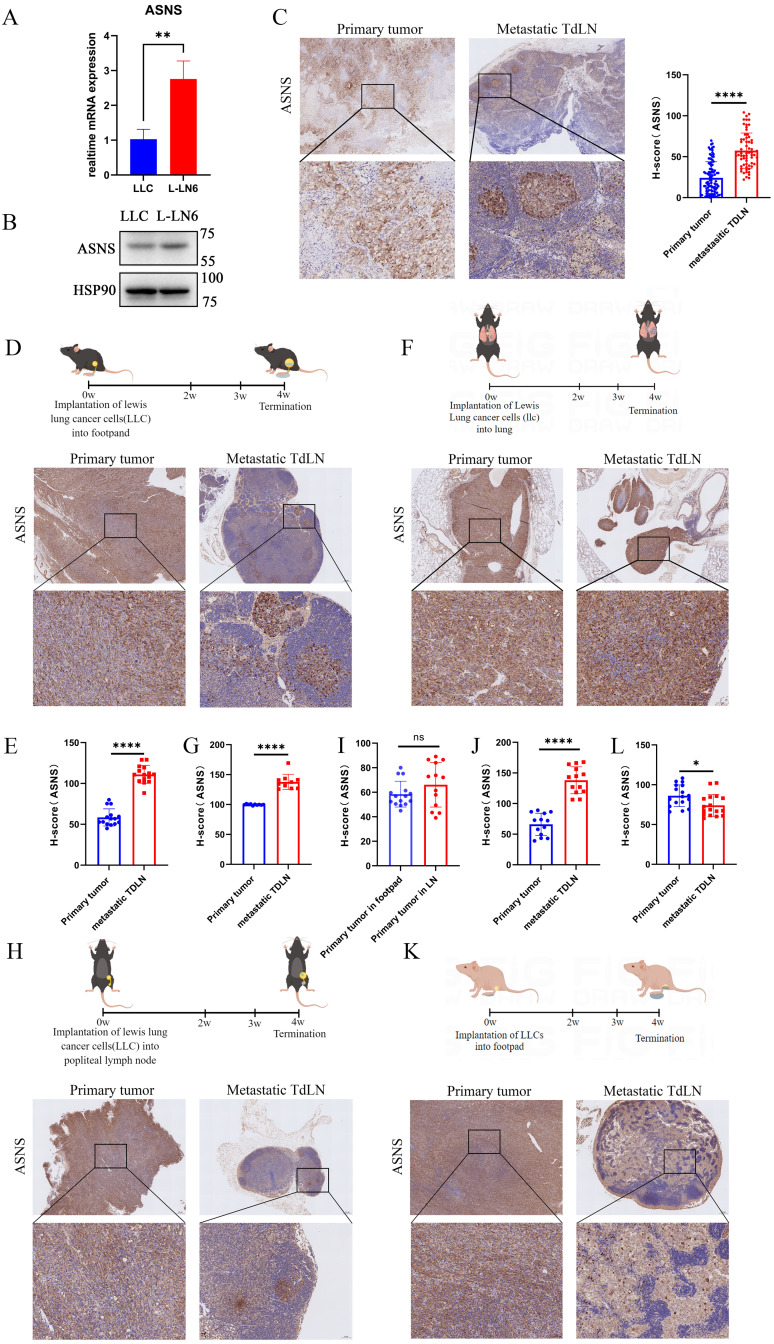
** Tumor cells with ASNS high expression were predisposed to metastasize in lymph nodes. Figure 2A**. RTPCR assays were used to detect the relative mRNA levels of ASNS in LLC and L-LN6. **2B**. Western blotting was used to evaluate the protein levels of ASNS in LLC and L-LN6. **2C**. Representative IHC images showing the expression of ASNS in primary tumor and LN metastasis from NSCLC patients (n=18). The ASNS expression between primary tumor and LN metastasis was analyzed with unpaired t test. **2D-E**. Schematics and representative IHC images showing the expression of ASNS in primary tumor and LN metastasis from the footpad implantation mouse model performed on C57BL/6 mice using LLC cells (n=6). The ASNS expression between primary tumor and LN metastasis was analyzed with paired t test. **2F-G**. Schematics and representative IHC images showing the expression of ASNS in primary tumor and LN metastasis from the intrapulmonary implantation mouse model performed on C57BL/6 mice using LLC cells (n=6). The ASNS expression between primary tumor and LN metastasis was analyzed with paired t test. **2H-J**. Schematics and representative IHC images showing the expression of ASNS in primary tumor and LN metastasis from the popliteal lymph node implantation mouse model performed on C57BL/6 mice using LLC cells (n=6). The ASNS expression between primary tumor in LN and in footpad or primary tumor and LN metastasis was analyzed with paired t test. **2K-L**. Schematics and representative IHC images showing the expression of ASNS in primary tumor and LN metastasis from the footpad implantation mouse model performed on nude mice using LLC cells (n=5). The ASNS expression between primary tumor and LN metastasis was analyzed with paired t test. The scale bars were indicated.

To prove the relationship between ASNS expression and LN metastasis, we initially compared ASNS expression in parental LLC cells and L-LN6. ASNS expression was markedly upregulated in L-LN6 at both the mRNA and protein levels (**Figure 2A-B**). Furthermore, we evaluated the ASNS expression of tumor cells *in situ* and metastatic LNs from NSCLC patients. The IHC staining of ASNS was markedly increased in tumor cells within LN metastases compared to those in the primary site (**Figure 2C**). In addition, to further validate the relationship in specific LN metastasis model and orthotopic lung cancer model, we used the murine lung cancer cell line LLC engineered to express the model antigen Ovalbumin (SIIINFEKL) to construct both footpad implantation and intrapulmonary implantation mouse models. Consistently, IHC examination of the primary tumors and TdLNs indicated a significant increase in ASNS protein expression in LN metastases (**Figure 2D-G**).

There were two potential mechanisms accounting for the elevated expression of ASNS protein in tumor cells within LN metastases, including that the subgroup with high ASNS expression within the primary tumor was predisposed to LN metastasis, or the microenvironment in LN including metabolic factors induced metabolic reprogramming in metastatic tumor cells to cause increased ASNS expression. To elucidate the underlying mechanism, LLC cells were injected into the popliteal LNs of C57/BL6 mice. Four weeks later, we harvested both the popliteal LNs (injection sites) and inguinal LNs (sites of metastasis) from the mice (**Figure 2H**). IHC staining revealed no enhancement of ASNS expression in tumor cells at the injection sites; however, a notable increase was observed in tumor cells within the metastatic sites (**Figure 2H-J**). The result indicated that the LN microenvironment did not influence the ASNS expression; rather, tumor cells with high ASNS expression exhibited a greater propensity for LN metastasis.

Then to verify whether the selection of tumor cell subsets with high ASNS expressing is immune-related, we utilized nude mice to establish the LN metastasis model. We observed an opposite trend in the disparity of ASNS expression in LN metastases compared to immunocompetent mice, implying a potential role of adaptive immunity (**Figure 2K-L**).

### ASNS enhances immunogenicity in lung cancer cells

**Figure 3:**
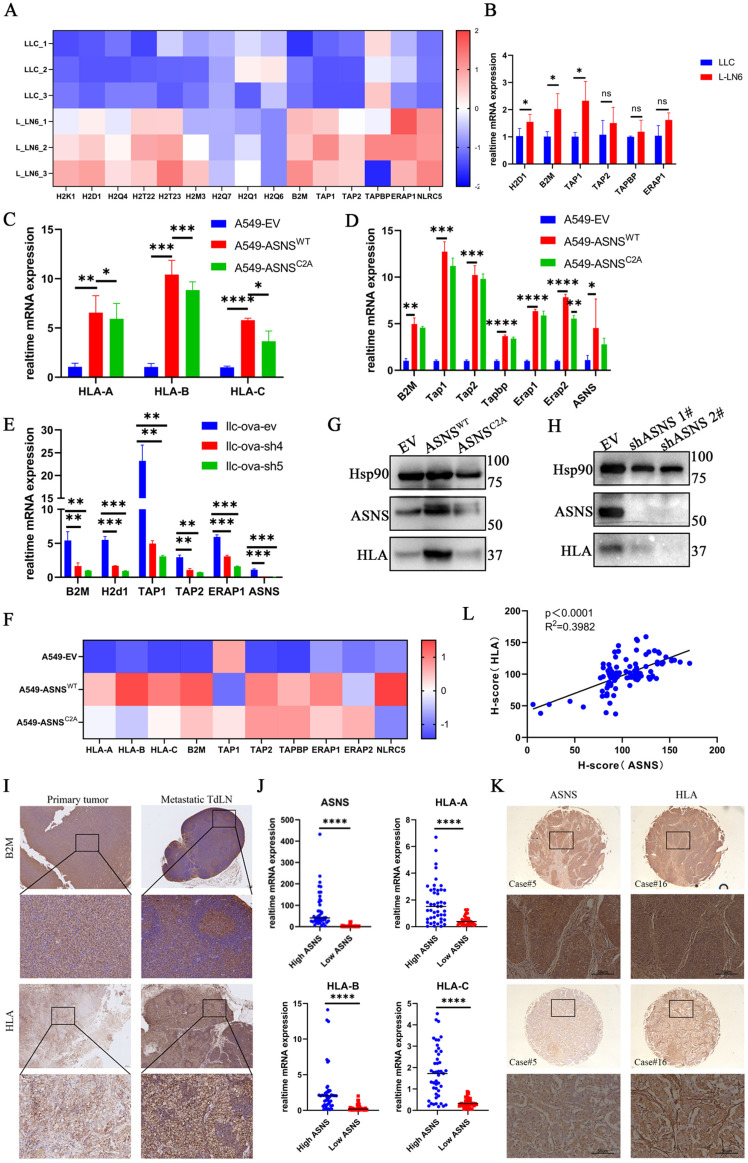
** ASNS enhances immunogenicity in lung cancer cells. Figure 3A**. Based on the RNA-seq datasets, the indicated gene expression is shown in LLC and L-LN6 (n=3).** 3B**. RT-PCR assays were used to detect the relative mRNA levels of antigen presentation genes in LLC and L-LN6. **3C**. RT-PCR assays were used to detect the effects of ASNS^WT^, ASNS^C2A^ and control group, over-expression on the induction of HLA mRNA level in A549 cells. **3D**. RT-PCR assays were used to detect the effects of ASNS^WT^ and ASNS^C2A^ over-expression on the induction of relative mRNA levels of antigen presentation genes in A549 cells.** 3E**. RTPCR assays were used to detect the effects of ASNS knockdown on the induction of relative mRNA levels of antigen presentation genes in LLC cells. **3F**. Western blotting was used to evaluate the effect of ASNS ^WT^ and ASNS^C2A^ over-expression on the induction of HLA protein level in A549 cells. **3G**. Western blotting was used to evaluate the effect of ASNS knockdown on the induction of HLA protein level in H1299 cells. **3H**. Based on the RNA-seq datasets, the indicated gene expression is shown in A549 cells with the overexpression of ASNS^WT^ and ASNS^C2A^ (n=3). **3I**. Representative IHC images showing the expression of HLA in primary tumor and LN metastasis from NSCLC patient (n=18) and B2M in footpad implantation mouse model performed on C57BL/6 mice (n=6). **3J**. The indicated gene expression in tumors from NSCLC patients was determined by qPCR (n=25). **3K-L**. Representative IHC images showing the expression of ASNS and HLA in primary tumor from two individual NSCLC patient. The correlation between ASNS expression and HLA expression was analyzed with linear regression (n=25).

It has been demonstrated that there is a significant upregulation of immunogenicity in LN-metastasis-specific melanoma cell lines, and this effect is independent of interferons (IFNs) 26. However, it remains unclear whether this phenomenon occurs in LN-metastasis-specific lung cancer cell lines and the underlying mechanisms driving it. To address this, we first demonstrated that, in the absence of IFN stimulation, immunogen-related pathway genes were upregulated at the RNA level in L-LN6 (**Figure 3A-B**). Given that the selection of ASNS-high-expression tumor cell subgroups in LN metastases is immune-related, we hypothesized that the elevated ASNS expression in LN metastasis sites may influence immune-related characteristics, including immunogenicity. Consequently, we explored the relationship between ASNS expression and the expression of genes involved in the IFN signaling pathway and antigen presentation 28. Previous studies have shown that the interferon markedly influenced IFN signaling pathway genes and antigen presentation pathway genes 26, 29-30. The low expression levels of related genes in tumor cells cultured *in vitro* without interferon stimulation possibly obscures the difference caused by ASNS expression level.

Therefore, we subcutaneously inoculated LLC cells with ASNS knockdown into C57/BL6 mice and then harvested tumor tissues to detect the expression of genes involved in the IFN signaling pathway and antigen presentation. The result showed that the expression of antigen presentation genes decreased in tumor tissues of the ASNS-knockdown group, while the expression of IFN-stimulated genes (ISGs) had no significant change (**Supplemental Figure 2A-B**).

To further support the regulatory effect of ASNS on immunogenicity, we further investigated whether ASNS expression in lung cancer cells, cultured *in vitro* with or without IFN treatment, could modulate immunogen-related pathway genes. We found that ASNS promoted the expression of immunogen-related pathway genes at the RNA level under IFN stimulation (**Figure 3C-F, Supplemental Figure 2C-F**). Additionally, we observed a slight, though statistically insignificant, increase in the expression of antigen presentation pathway genes in ASNS^WT^-overexpressed cells without treatment of IFN-γ. Furthermore, ASNS could enhance the expression of immunogen-related pathway genes at the protein level in the absence of interferon stimulation (**Figure 3G-H**).

Moreover, to identify whether the metabolic function of ASNS influenced its regulation of immunogen-related pathway genes, we utilized the constructed ASNS mutated cell lines (ASNS^C2A^) wherein the coding protein was unable to catalyze the synthesis of asparagine 20, 31. Our results demonstrated that overexpression of ASNS^C2A^ could enhance the expression of immunogen-related pathway genes at the RNA level, albeit to a lesser extent than overexpression of ASNS^WT^ (**Figure 3C-F, Supplemental Figure 2C-E**). Besides, ASNS^C2A^ failed to enhance the protein-level expression of genes associated with immunogenic pathways (**Figure 3G**). This suggested that the metabolic function of ASNS might play an essential role in its regulatory effect on antigen presentation pathway genes.

To validate the aforementioned findings through *in vivo* experiments, we assessed the immunogenicity in primary tumor tissues and metastatic LN tissues from both NSCLC patients and mouse models. The results indicated a consistent trend in immunogenicity associated with ASNS (**Figure 3I, Supplemental Figure 3A**). Furthermore, mRNA and protein level analysis in 25 paired lung cancer tissues and IHC examination of lung cancer tissue arrays revealed a positive correlation between HLA expression and ASNS expression (**Figure 3J-K**). Together, these results demonstrated that ASNS could modulate the expression of immunogen-related pathway genes, with its regulatory effect being dependent on its metabolic function.

### ASNS promotes alpha-aminobutyric acid secretion to enhance the immunogenicity of lung cancer cells and the cytotoxicity of CD8+ T cells *in vitro*

**Figure 4:**
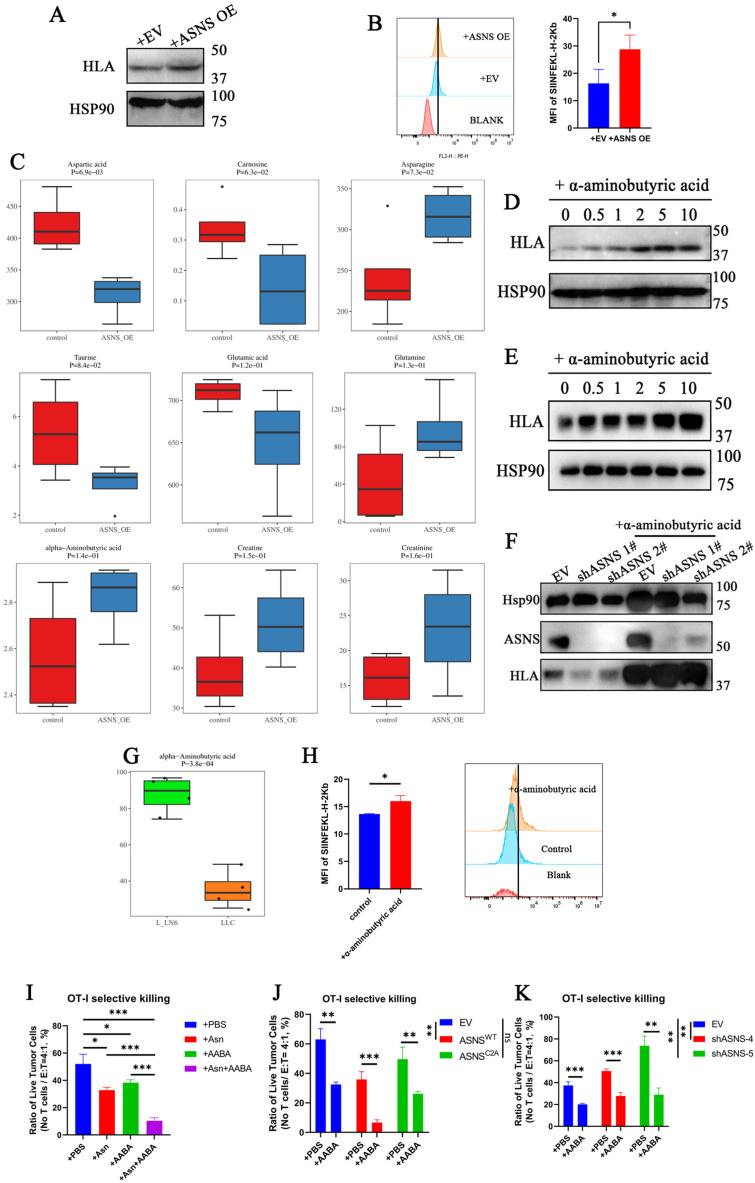
** ASNS promotes alpha-aminobutyric acid secretion to enhance MHC expression of lung cancer cells and the cytotoxicity of CD8+ T cells. Figure 4A**. Western blotting was used to determine the expression of HLA upon the treatment of culture medium supernatant from A549 cells with the overexpression of ASNS^WT^ and EV for 24 hours. **4B**. Histogram showing the expression levels of SIINFEKL-H-2Kb in LLC-OVA cells upon the treatment of culture medium supernatant from LLC-OVA cells with the overexpression of ASNS^WT^ and EV for 24 hours. **4C**. Metabolomics analysis was performed on the culture medium supernatant of A549 cells with the overexpression of ASNS^WT^ and the control group. **4D-E**. A549 cells(C) and H1299 cells(D) were treated with α-aminobutyric acid for 48 h. The expression of HLA was determined by western blotting. **4F**. Western blotting were used to detect the effects of ASNS knockdown on the induction of HLA protein level upon or not the treatment of α-aminobutyric acid for 48 h. **4G**. Metabolomics analysis of alpha-aminobutyric acid was performed on LLC-parental cells and LLC-LN6 cells. **4H**. Histogram showing the expression levels of SIINFEKL-H-2Kb in LLC-OVA cells with or without α-aminobutyric acid treatment for 48 h. Quantitative estimates of SIINFEKL-H-2Kb levels in LLC-OVA cells (n=3). **4I-J**. LLC-Ova cells with the overexpression of ASNS ^WT^ and ASNS^C2A^ (I) and knockdown of ASNS (J) were mixed with OT-I CD8+T cells at a 4:1 ratio, and (H)tumor cells viability was evaluated (n=3).

Due to that ASNS functions as a metabolic enzyme and the overexpression of ASNS^C2A^ results in a significantly weaker upregulation of immunogenicity compared to ASNS^WT^, we proposed that ASNS regulated immunogenicity through the secretion of small-molecule metabolites. To validate the hypothesis, we cultured lung cancer cells in the supernatant of the culture medium derived from the same cell line overexpressing ASNS^WT^ or the control group separately. The results showed that the culture medium from the ASNS^WT^-overexpressing group notably enhanced the immunogenic expression of lung cancer cells (**Figure 4A**). This finding was further corroborated by utilizing the supernatant of the culture medium derived from the same cell line overexpressing ASNS^WT^ to stimulate LLC-OVA, the murine lung cancer cell line expressing the tumor mimetic antigen ovalbumin. Flow cytometry analysis revealed an increase in H-2Kb-SIINFEKL (MHC-OVA) expression without interferon stimulation *in vitro* (**Figure 4B**). Given that asparagine (Asn) is the principal product of ASNS, we initially conducted *in vitro* experiments involving Asn deprivation and Asn stimulation. Regrettably, Asn was not found to be the crucial metabolite playing a role in immunogen upregulation (**Supplemental Figure 3B-C**).

To further investigate the hypothesis, metabolomics analysis was performed on the supernatant of culture medium derived from tumor cells overexpressing ASNS^WT^ and the control group. The analysis revealed significant increases in α-aminobutyric acid (AABA), creatine, and creatinine, in addition to asparagine (**Figure 4C**).

Subsequently, these identified differential metabolites were employed to stimulate lung cancer cell lines, and only α-aminobutyric acid markedly upregulated the immunogenicity (**Figure 4D-E, Supplemental Figure 3D-E**). Furthermore, the decrease in immunogen expression resulting from ASNS knockdown could be compensated by treatment with AABA (**Figure 4F**). Further, we analyzed the metabolomics data of LN6 cells and validated the consistent elevation in AABA in murine LN metastatic tumor lines (**Figure 4G**). Flow cytometry analysis revealed AABA can also increase the H-2Kb-SIINFEKL (MHC-OVA) expression in LLC-OVA without interferon stimulation *in vitro* (**Figure 4H**). To explore its immunomodulatory effects to CD8+ T cells, we treated OT-I CD8⁺ T cells with Asn, AABA, or a combination of both. Asn treatment enhanced CD8⁺ T cell activation, whereas AABA alone had no significant effect (**supplemental 3F**). When OT-I CD8⁺ T cells were co-cultured with LLC-OVA tumor cells in the presence of individual metabolites, both Asn and AABA independently enhanced antigen-specific cytotoxicity. Notably, their combined application elicited a synergistic enhancement of T cell-mediated tumor cell killing (**Figure 4I and supplemental 4A**). These findings suggest that AABA cooperatively promotes anti-tumor immunity by enhancing tumor immunogenicity rather than directly promoting CD8+ T cell activation. Additionally, tumor cells with higher ASNS expression were observed to be more susceptible to antigen-specific CD8+T cell killing, indirectly verifying the upregulation effect of ASNS on immunogenicity (**Figure 4J-K and supplemental 4B-C**). And the impaired cytotoxic function of antigen-specific CD8⁺ T cells resulting from low ASNS expression was partially rescued by treatment with AABA (**Figure 4J-K and supplemental 4B-C**). To evaluate the immunomodulatory effects of AABA *in vivo*, we developed the LN metastasis mouse models treated with either AABA or PBS control. In comparison to the PBS-treated group, the AABA-treated group exhibited significant inhibition of tumor growth and reduction in tumor volume, especially in EV and ASNS^C2A^ groups (**Supplemental Figure 6**). In conclusion, these results suggested that ASNS promoted α-aminobutyric acid secretion to enhance the immunogenicity of lung cancer cells and the cytotoxicity of CD8+ T cells.

### ASNS reshapes the immune landscapes in metastatic TdLNs and primary tumor sites

Owing to the pivotal role of TdLNs to moderate immune editing in primary tumor, the immune landscape of TdLNs represents a critical determinant of both prognosis and therapeutic responsiveness. Recent studies have identified tumor-antigen specific stem-like CD8+T (Tsl) cells as the primary responders of ICB treatment, with these cells being predominantly reserved in TdLNs. However, prior investigations have largely focused on TdLNs without metastasis, leaving the phenotypic and functional characteristics of CD8⁺ T cells in metastatic TdLNs poorly understood. Our previous work revealed that LN metastasis facilitates tumor-specific antigen presentation in TDLNs, leading to robust T cell activation and memory formation, and that subsequent immune remodeling in the primary tumor. Building on these findings and the above results, we next examined the immune landscape of metastatic TdLNs with a particular focus on T cell stemness, and the potential regulatory function of ASNS expression on immune remodeling. First, we constructed the LN metastasis mouse models utilizing LLC stable cell line with ASNS overexpression or ASNS knockdown. Due to that gene knockdown in stable cell lines inevitably underwent off-target effects and cancer cells with ASNS high expression were predisposed to metastasize into LNs, the metastatic tumor cells in TdLNs of the ASNS knockdown group did not show significantly lower expression compared to the control group (**Supplemental Figure 5**). Consequently, our subsequent experiments utilized stable cell lines with overexpressed ASNS.

**Figure 5:**
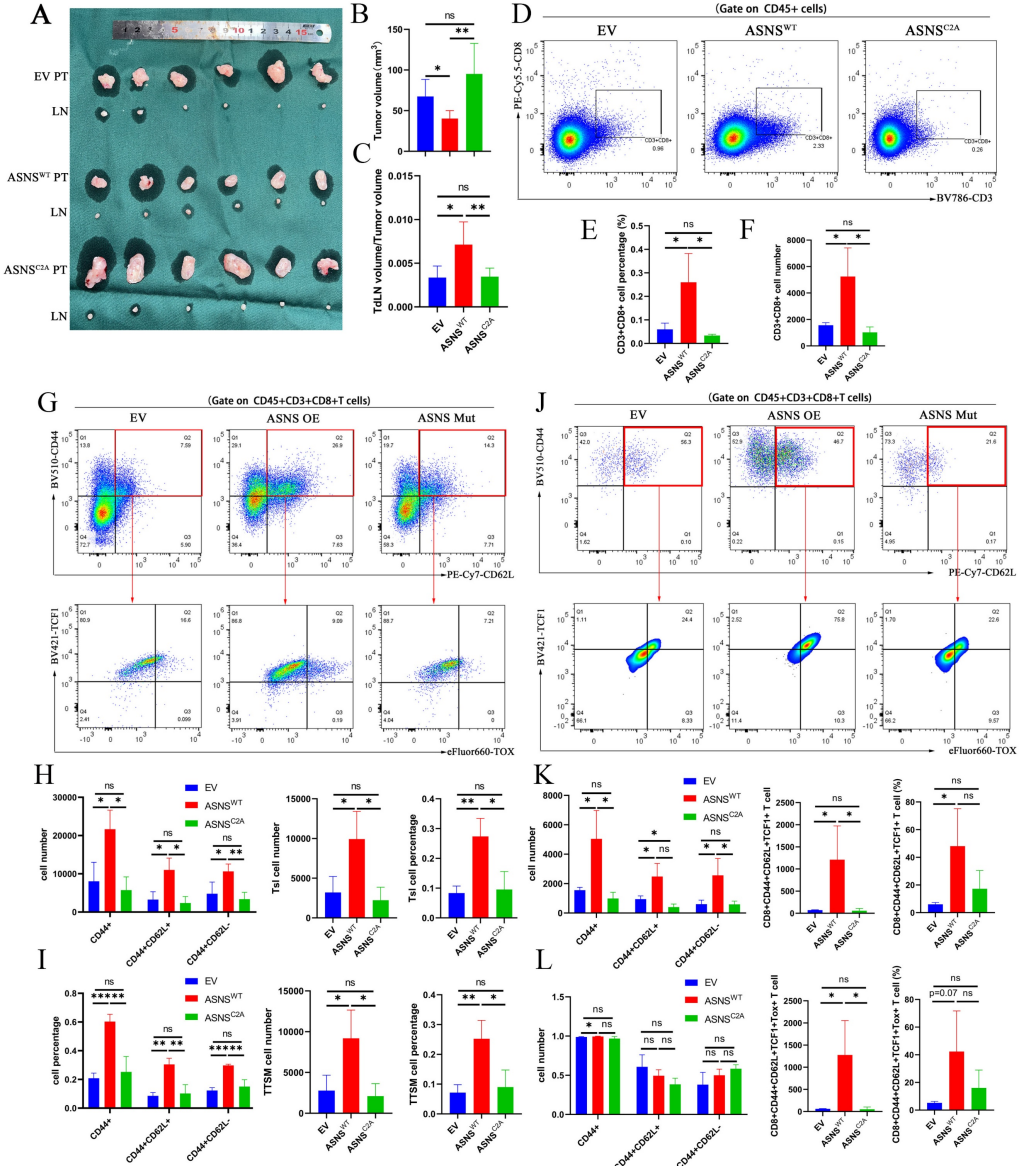
** ASNS shapes the immune landscapes in metastatic TdLN and primary tumor site. Figure 5A-C**. LN metastasis model was conducted on C57BL/6 mice with LLC-ASNS^ WT^(n=6), ASNS^C2A^ overexpression cells(n=6) and control group(n=6), primary tumor and popliteal lymph nodes were isolated at the end of the experiment, and primary tumor volume(B) and TdLN volume/tumor volume(C) was measured and analyzed. **5D-F**. (D) Representative FACS profiles of CD8+T cells are shown. The percentage(E) and number(F) of CD8+ subset in TIL cells isolated from primary tumor is shown. **5G-I**. (G) Representative FACS profiles of the co-expression pattern of CD44 and CD62L, or the co-expression pattern of TCF-1and TOX in CD8+ T cells are shown. The number(H) and percentage(I) of CD44+, CD44+CD62L+, CD44+CD62L-, Tsl and TTSM subset in CD8+T cells isolated from TdLN is shown. **5J-L**. (J) Representative FACS profiles of the co-expression pattern of CD44 and CD62L, or the co-expression pattern of TCF-1and TOX in CD8+ T cells are shown. The number(K) and percentage(L) of CD44+, CD44+CD62L+, CD44+CD62L-, Tsl and TTSM subset in CD8+T cells isolated from primary tumor is shown.

Through constructing the LN metastasis mouse models, we observed that overexpressed ASNS^WT^ resulted in a reduction in tumor volume and an augmented ratio of metastatic TdLNs to tumor volume (**Figure 5A-C**). Flow cytometry analysis conducted on the primary tumors revealed a notable increase in CD8+ T cell infiltration upon ASNS^WT^ overexpression (**Figure 5D-F**). ASNS was a key enzyme for the production of asparagine (**Figure 4B**), and asparagine was a crucial metabolite to potentiate CD8+ T cell activation and memory 21-22. Therefore, we analyzed the activation and memory of CD8+ T cells in the metastatic TdLNs collected from mouse models.

Our results showed an obvious enhancement in both activation (CD44+) and memory (CD44+CD62L+) of CD8+T cells (**Figure 5G-I**). Stem-like CD8+T cells (Tsl, CD44+TCF1+) have recently been identified as crucial immunocytes for sustaining long-term cancer immunity and responding to immunotherapy, persisting in TdLNs throughout disease progression 14-15. Subsequent researches have further characterized the features of Tsl cells and identified this T cell subgroup as TdLN-derived stem-like and memory T cells (TTSM cells, CD44+CD62L+TCF1+Tox-) in detail 16. To determine whether ASNS influenced the distribution of Tsl and TTSM cells, we analyzed the expression of TCF1 and TOX on CD44+CD62L+CD8+T cells and found the enrichment of Tsl and TTSM cells in TdLNs from overexpressed ASNS^WT^ group than that from overexpressed ASNS^C2A^ group or control group (**Figure 5G-I**).

Then to assess immune remodeling at the primary tumor site, we quantified T-cell infiltration and differentiation using flow cytometry. The results revealed a significant increase in the number of activated, memory, and stem-like CD8+T cells in the primary tumor tissue harvested from the overexpressed ASNS^WT^ group compared to the overexpressed ASNS^C2A^ group or control group, though the proportion was not significantly different (**Figure 5J-L**). In conclusion, ASNS in tumor metastasis could promote T cell activation and memory, as well as enrichment of Tsl and TTSM cells in metastatic TdLNs, to further regulate the immune remodeling at the primary tumor sites.

### ASNS-high-expression tumor metastases generate lymphocyte niches enriched with activated T cells, memory T cells, Tsl, and TTSM

**Figure 6:**
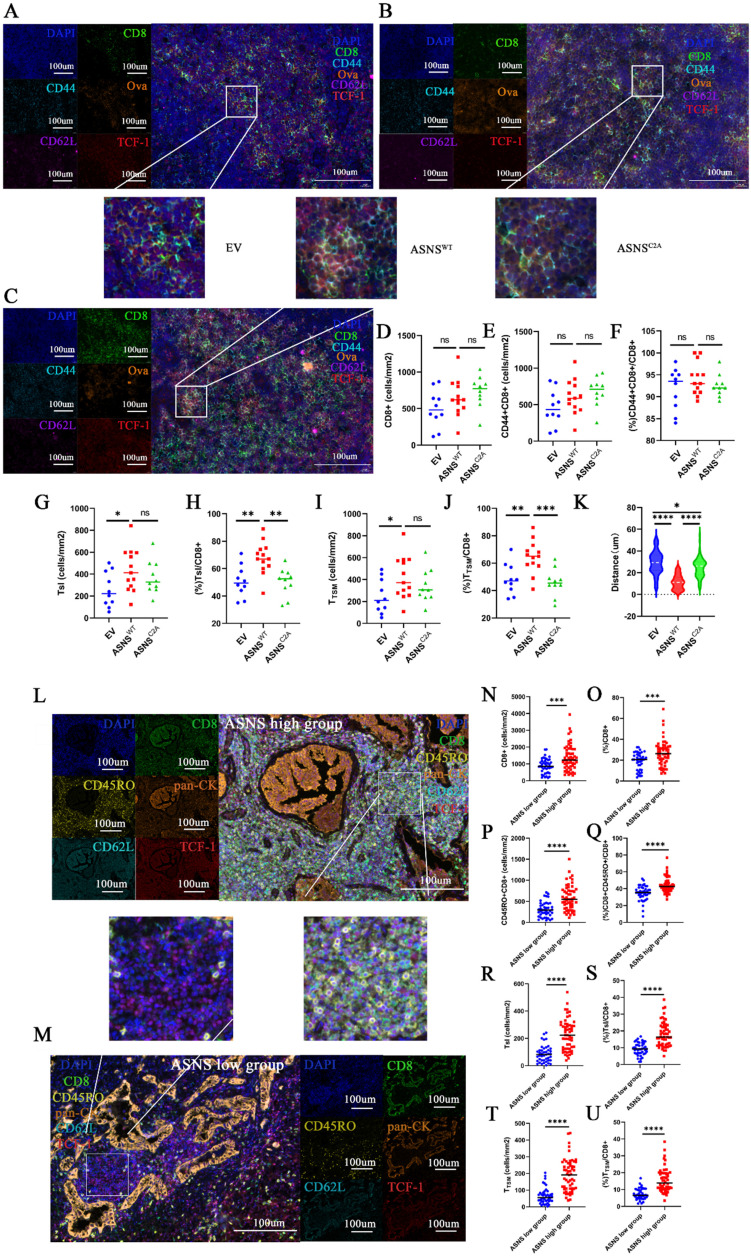
** ASNS-high-expression metastases generated lymphocyte niches enriched with activated T cells, memory T cells, Tsl and TTSM. Figure 6A-C**. Representative immunofluorescence staining images of metastatic TdLNs from LN metastasis model. **6D**. The number of CD8+ T cells in the metastasis locations within TdLNs (ASNS^WT^, n=6, ASNS^C2A^, n=5, and EV, n=4). **6E-F**. The number(E) and percentage(F) of CD44+CD8+T cells among all CD8 T cells in the metastasis locations within TdLNs (ASNS^WT^, n=6, ASNS^C2A^, n=5, and EV, n=4).** 6G-H**. The number(G) and percentage(H) of Tsl cells among all CD8 T cells in the metastasis locations within TdLNs (ASNS^WT^, n=6, ASNS^C2A^, n=5, and EV, n=4).** 6I-J**. The number(I) and percentage(J) of TTSM cells among all CD8 T cells in the metastasis locations within TdLNs (ASNS^WT^, n=5, ASNS^C2A^, n=4, and EV, n=3).** 6K**. Quantitative estimates of the distance from ova+ to CD8+CD44+CD62L+TCF+(TTSM) (ASNS^WT^, n=6, ASNS^C2A^, n=5, and EV, n=4). **6L-M**. Representative immunofluorescence staining images of metastatic TdLNs from NSCLC patients. **6N-O**. The number(C) and percentage(D) of CD8+ T cells in the metastasis locations within TdLNs(ASNS high group, n=7, and ASNS low group, n=6). **6P-Q**. The number (E) and percentage(F) of CD45RO+CD8+ T cells in CD8+T cells in the metastasis locations within TdLNs(ASNS high group, n=7, and ASNS low group, n=6).** 6R-S**. The number (G) and percentage(H) of Tsl cells in CD8+T cells in the metastasis locations within TdLNs(ASNS high group, n=7, and ASNS low group, n=6).** 6T-U**. The number (I) and percentage(J) of TTSM cells in CD8+T cells in the metastasis locations within TdLNs(ASNS high group, n=7, and ASNS low group, n=6).

Then, we sought to elucidate the interaction and spatial relationship between tumor cells with varying levels of ASNS expression and CD8+T cells in metastatic TdLNs. We conducted fluorescent multiplex IHC (mIHC) on metastatic LNs harvested from LN metastasis mouse models (**Figure 6A-C**). Congruent with the results of flow cytometry analysis, the distribution of CD8+ T cells was notably higher surrounding tumor metastases in TdLNs from the overexpressed ASNS^WT^ group than that from the overexpressed ASNS^C2A^ group or control group (**Figure 6D**). Among the CD8+ T cells, the proportion of activated T cells (CD8+CD44+), Tsl (CD8+CD44+TCF-1+) cells, and TTSM (CD8+CD44+CD62L+TCF-1+) cells were significantly higher in the vicinity of tumor metastases in TdLNs from overexpressed ASNS^WT^ group compared to the overexpressed ASNS^C2A^ group or control group (**Figure 6E-J**). As that tumor metastasis modulated differentiation of T cells by Asn secretion required proximity to the cognate T cell, we analyzed the distance from tumor metastasis (ova+) to TTSM (CD8+CD44+CD62L+TCF+) in TdLNs and found that the distance from overexpressed ASNS^WT^ group was shorter than other two groups (**Figure 6K**). In summary, our data suggested that the tumor metastases with ASNS^WT^ overexpression had a more significant impact on promoting activation and memory of surrounding CD8+T cells, as well as enrichment of Tsl and TTSM populations.

Single-cell RNA sequencing (scRNA-seq) analysis conducted on TdLN tissues from NSCLC patients has revealed the presence of TTSM cells and proved this subgroup as bona fide responders to PD-1/PD-L1 blockade treatment in TdLNs 16-17. However, the existence, spatial distribution, and correlation with ASNS of this population within human metastatic TdLNs remained unclear. Thus, we firstly utilized scRNA-seq datasets (GEO: GSE131907) of CD8+ T cells in TdLNs from patients with lung adenocarcinoma 32. Totally, 9216 cells from 16 samples were further categorized into six clusters including TTSM (**Supplemental Figure 9A-C**) 16. Then we observed a similar proportion of TTSM compared with non-metastatic TdLNs, despite the increased proportion of exhausted T cells and the reduced presence of naïve T cells in metastatic TdLNs (**Supplemental Figure 9D-E**). Furthermore, to explore the spatial distribution of TTSM in human metastatic TdLNs, we performed mIHC on metastatic TdLNs collected from NSCLC patients and stratified the samples into two groups based on the ASNS expression of the primary tumor (**Figure 6L-M, Supplemental Figure 10**). As anticipated, the abundance of CD8+ T cells was observed to be higher surrounding tumor metastases in TdLNs of lung cancer patients from the ASNS high group than that from the ASNS-low group (**Figure 6N-O**). In alignment with results on metastatic TdLNs in mouse models, among the CD8+ T cells, the proportion of activated T cells (CD8+CD45RO+), Tsl (CD8+CD45RO+TCF-1+) cells, and TTSM (CD8+CD45RO+CD62L+ TCF-1+) cells were significantly elevated in the vicinity of tumor metastases in TdLNs of lung cancer patients from ASNS-high-group in contrast to that from ASNS-low-group (**Figure 6P-U**).

These results disclosed that tumor metastases with high ASNS expression were associated with more active immune environments, characterized by upregulated immune activation, memory, and a higher concentration of Tsl and TTSM cells, compared to metastases with low ASNS expression, which suggested that tumor metastases with high ASNS expression promoted the development of lymphocyte niches exhibiting enhanced immune activation, memory, and enrichment of Tsl and TTSM cells.

### ASNS was correlated with both survival outcomes and the response to PD-1-based neoadjuvant immunotherapy in NSCLC patients with LN metastases

Our data from both mice and humans uncovered that ASNS enhanced the immunogenicity of lung cancer cells and reshaped the immune microenvironment in metastatic TdLNs to modulate CD8+T cell infiltration in the tumor *in situ*. To evaluate the predictive value of ASNS in NSCLC patients, we analyzed the overall survival (OS) and disease-free survival (DFS) based on the TCGA dataset of NSCLC, LUSC, and LUAD respectively. We observed that high expression of ASNS correlated with better prognosis in NSCLC patients, as evidenced by a slight trend of longer OS in the TCGA dataset for LUSC although it did not reach statistical significance, as well as longer DFS in the TCGA dataset for NSCLC and LUAD (**Supplemental Figure 11A-F**). Furthermore, we validated our findings above using TCGA data of NSCLC and demonstrated that ASNS expression is positively correlated with the expression of effector markers of CD8+T cells (GZMA, GZMB, and IFNG) (**Supplemental Figure 11G-J**), lymphocyte infiltration signature score, CD8+T Cells infiltration signature score, cytolytic index, and immune score (**Supplemental Figure 11K-N**). Additionally, bioinformatic analysis using TIMER2.0 consistently showed a significantly positive correlation between ASNS expression and the immune infiltration level of memory T cells (**Supplemental Figure 11O**).

We next wondered whether ASNS-induced remodeling of the TME and metastatic TdLN microenvironment worked in patients treated with immunotherapy. A total of 18 patients with NSCLC were enrolled from April 30, 2023, to December 31, 2024 (**Supplemental Figure 12**). Among 18 recruited patients, the majority (72.2%) were with squamous cell carcinoma; 16 patients (88.9%) were with stage IIA to IIIA disease (as per the TNM Classification for Lung Cancer, IASLC, ninth edition), while 2 patients were with resectable stage IIIB, including one with stage T4N2M0, and another with T4N3M0. Most patients (77.7%) had a history of smoking, and 18 patients (100.0%) were with LN involvement (**Table 1**). By December 31, 2024, 18 patients (100.0%) had received three cycles of anti-PD-1-based neoadjuvant therapy and subsequently underwent surgical resection.

**Figure 7:**
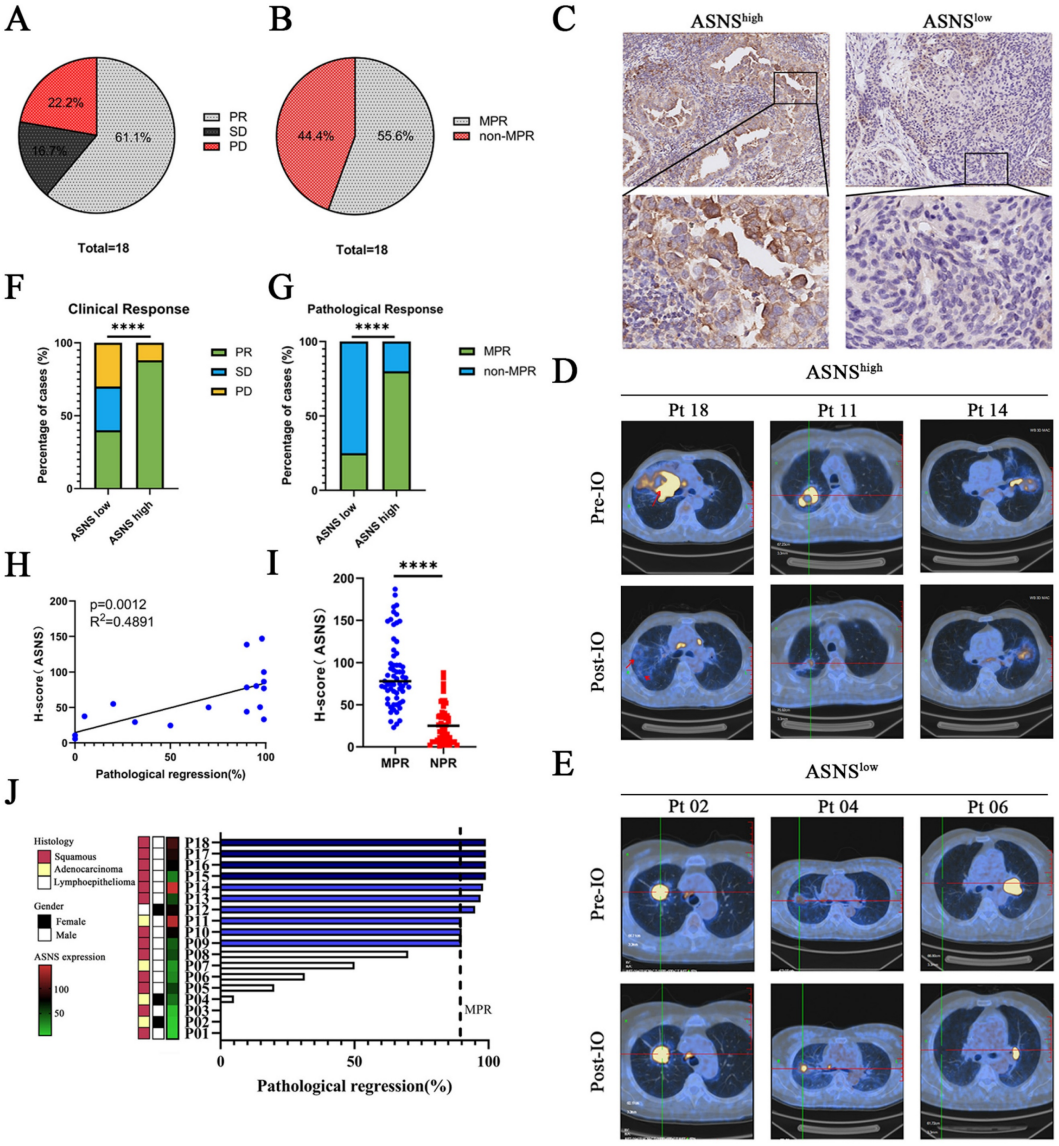
** ASNS-expression of human lung cancer with LN metastasis impacts neoadjuvant anti-PD-1 efficacy. Figure 7A**. Clinical outcome assessments of the cohort of 18 patients with resectable NSCLC treated with anti-PD-1-based neoadjuvant immunotherapy combined with chemotherapy. The ORR was 61.1% (11 of 18 patients), and the DCR was 83.3% (15 of 18 patients). 11 patients (61.1%) had partial responses (PR), 4 patients (22.2%) had stable disease (SD), and 3 patients (16.7%) had progressive disease (PD). Best change from baseline in the tumor burden per patient referring to the modified RECIST (mRECIST) guidelines.** 7B**. Pathological response assessments of the cohort of 18 patients with resectable NSCLC treated with anti-PD-1-based neoadjuvant immunotherapy combined with chemotherapy. The MPR was 55.6% (10 of 18 patients), and the DCR was 44.4% (8 of 18 patients). **7C**. Immunohistochemistry (IHC) staining of ASNS in NSCLC tumors that received neoadjuvant immunotherapy. IHC plots represent ASNS^high^ tumors (upper) and ASNS^low^ tumors (bottom). **7D-E**. Representative patients with resectable NSCLC who received neoadjuvant anti-PD-1 therapy and then treated with surgery. Representative PETCT scans of target lesions. **7F-G**. Comparisons of clinical response (E) and pathological response (F) between ASNS^low^ (n = 11 patients) and ASNS^high^ groups (n = 7 patients). **7H.** The correlation between ASNS expression and pathological regression was analyzed with linear regression (n = 18). **7I.** Comparisons of the ASNS expression between MPR group and NPR group was analyzed with unpaired t test. **7J**. Percentage of pathologic regression. Pathological regression is defined as percentage viable tumor cells - 100%. PR means partial response; SD means stable disease; PD means progression of disease, MPR means major pathological regression.

Among 18 patients with evaluable radiographic responses, 11 (61.1%) experienced a partial radiological response, yielding an overall response rate (ORR) of 61.1%. Additionally, 3 patients (16.7%) achieved stable disease, contributing to a disease control rate (DCR) of 77.8%, while 4 patients (22.2%) showed progressive disease (**Figure 7A, Table 1**). Among 18 patients with evaluable surgical tumor samples, 10 (55.5%) achieved major pathological response (MPR), including 8 with squamous cell carcinoma, 1 with adenocarcinoma, and 1 with lymphoepithelioma (**Figure 7B, Table 1**). The remaining 8 patients (44.5%) did not achieve MPR, comprising 5 with squamous cell carcinoma and 3 with adenocarcinoma (**Figure 7B, Table 1**).

18 patients who underwent neoadjuvant immunotherapy were categorized into two groups based on the IHC analysis of ASNS expression in resected tumor specimens. IHC analysis identified 10 patients exhibiting low ASNS expression (IHC -/+) and 8 patients exhibiting high ASNS expression (IHC ++/+++) (**Figure 7C**). Radiographic findings revealed a notable disparity in clinical response between the two groups (**Figure 7D-F**). Among the 11 patients with low ASNS expression, 4 (40%) exhibited a partial response (PR), 3 (30%) had stable disease (SD), and 3 (30%) experienced disease progression (PD). In contrast, 7 (87.5%) of the 8 patients in the high ASNS expression group achieved PR, while 1 (12.5%) had disease progression (PD) (**Figure 7F**). Furthermore, ASNS expression in tumor cells at the primary tumor site was positively correlated with the percentage of pathological response (**Figure 7G-J**). Together, these data confirmed the association between ASNS expression and the efficacy of anti-PD-1-based neoadjuvant immunotherapy in NSCLC patients with LN metastases.

**Figure 8:**
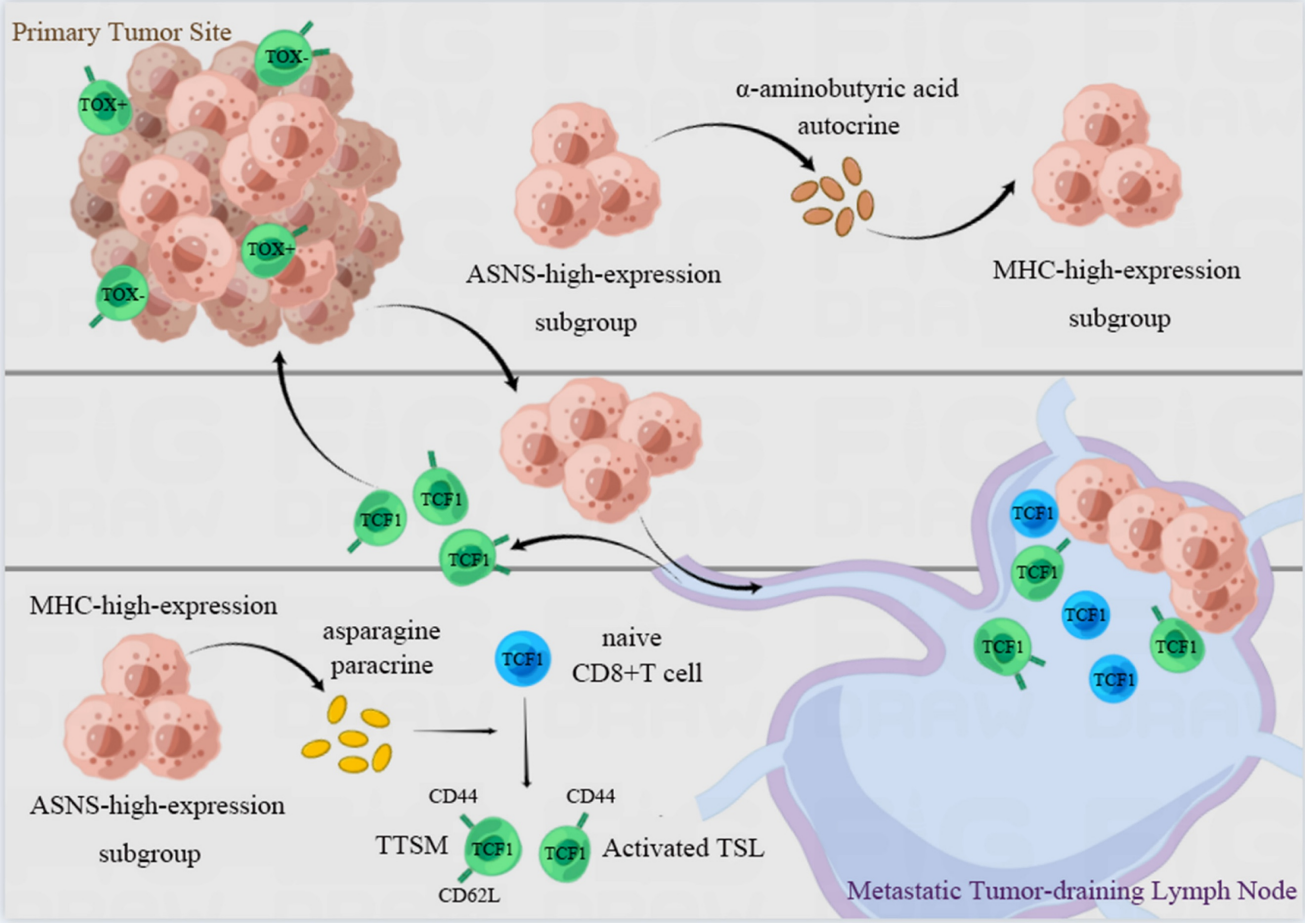
**Illustration of the proposed working model.** Lung cancer cells with ASNS high expression were predisposed to metastate in lymph nodes. ASNS facilitated α-aminobutyric acid secretion to augment both the immunogenicity of lung cancer cells and the cytotoxicity of CD8+ T cells. And LN metastasis metastases characterized by high ASNS expression establish lymphocyte-supportive niches that facilitate T cell activation, memory, and the generation of TSL and TTSM populations, particularly in the vicinity of metastatic foci. This process is orchestrated by paracrine asparagine release and augmented tumor immunogenicity mediated by α-aminobutyric acid, which together reshape the immune microenvironment at both the primary tumor site and within metastatic lymph nodes. This immune reprogramming enhances the therapeutic efficacy of neoadjuvant immunotherapy in NSCLC patients with lymph node involvement.

In the present study, we have verified that lung cancer cells with high ASNS expression were predisposed to metastasize in LNs. ASNS facilitated α-aminobutyric acid secretion to augment both the immunogenicity of lung cancer cells and the cytotoxicity of CD8+ T cells *in vitro*. Furthermore, LN metastasis with ASNS high expression fosters the development of lymphocyte niches permissive for T cell activation and memory, as well as the production of TSL and TTSM cells in metastatic LNs, especially in the vicinity of metastatic foci. This process reshaped the immune microenvironment in both the primary tumors and metastatic LNs, ultimately enhancing the efficacy of neoadjuvant immunotherapy in NSCLC patients with LN metastasis (**Figure 8**).

## Discussion

In recent years, immune checkpoint blockade (ICB) has revolutionized cancer therapy, with neoadjuvant ICB now recognized as a crucial component of the multidisciplinary management of resectable NSCLC, particularly in advanced stages with LN metastases. This approach has significantly improved overall survival and facilitated durable tumor control 9, 33-42. Besides, neoadjuvant therapies offer a unique opportunity to investigate the genetic signatures associated with responses to ICB treatment in patient cohorts. More importantly, the abundant availability of tumor tissues and dissected TdLNs, facilitated by advances in surgical procedures for NSCLC, provides a valuable opportunity to investigate the mechanisms underlying both resistance and responsiveness to immunotherapy 43-47. Consequently, further research should focus on understanding the mechanisms through which metastatic TdLNs influence immune environment remodeling and the subsequent impact on responses to neoadjuvant immunotherapy.

Here, we reported that high ASNS expression was one of the characteristics of lung cancer cells in LN metastasis, which played an essential role in the interaction between immunocytes and tumor cells within metastatic TdLNs. We found that ASNS promoted alpha-aminobutyric acid secretion to enhance immunogenicity of lung cancer cells independently of IFN treatment, which could exactly elucidate that upregulation of MHC expression in LN-metastasis-specific cell lines (LN line) independently of exposure to IFNs 26. However, owing to limited current knowledge of α-aminobutyric acid and our financial constraints, the mechanistic link between ASNS and α-aminobutyric acid remains to be further explored.

It has been reported that TdLNs served as reservoirs of TTSM cells to sustain antitumor T cells persistently during tumor development and protect them from the terminal differentiation in the TME 14-17. To further investigate whether TTSM cells were similarly enriched in metastatic TdLNs and the impact of ASNS on the process, we demonstrated that lung cancer cells with high ASNS expression enhanced CD8+T cells activation, memory, as well as generation of Tsl and TTSM cells in metastatic TdLNs, and sequentially promoted infiltration of CD8+T cells in primary tumor sites to control tumor growth. Besides, the significantly remodeled immune environments were observed in the vicinity of tumor metastasis, especially in the overexpressed ASNS^WT^ group, while T cells distant from the tumor predominantly remained in the naive state, suggesting that tumor metastases with high ASNS expression generated lymphocyte niches enriched with activated T cells, memory T cells, Tsl and TTSM cells through asparagine paracrine 21-22. However, previous studies and *in vitro* experiments have suggested that both ASNS-overexpressed tumor cells and LN lines could promote CD8+T cell differentiation into effector T cells instead of TTSMs *in vitro* or primary tumor sites 23,26. Together, tumor metastasis with high ASNS expression promoted T cell activation and memory within TdLNs, and there was an unknown underlying mechanism within LNs that maintained CD8+ T cell stemness, possibly contributing to immune evasion in metastatic TdLNs. Recent researches on cellular interactions and molecular pathways regulating CD8+ T cell stemness maintenance and differentiation have demonstrated that cell factor including TGF-β, TCR engagement, CD28 co-stimulation and several metabolites such as IPA, lactate, L-Arginine, extracellular hydrogen ion, extracellular potassium ion and Vitamin A epigenetically or transcriptionally regulating the expression of TCF7 and other stem-like T cell related genes to modulate T cell stemness 18,48-55. Further studies have also confirmed that fibroblastic reticular cell niches in TdLNs also play a crucial role in maintaining the stemness of CD8+T cells 56-57. Nevertheless, the potential mechanisms underlying LN metastasis still need to be further investigated.

Recent researches have uncovered that TTSM cells exhibited optimal anti-tumor activity in immune checkpoint blockade treatment in highly immunogenic tumors 58. Furthermore, multifactorial mechanisms of sensitivity and resistance suggested that biomarkers such as PD-L1 or tumor mutational burden (TMB) cannot solely explain the response to immunotherapy. This implied that biomarkers associated with various stages of the cancer-immune (CI) cycle were necessary to investigate 59-60. In conjunction with our findings above, we were surprised to discover that ASNS enhanced the anti-tumor immune by impacting both the immunogenicity of lung cancer cells in the primary tumor and the proportion of TTSM in metastatic TdLNs. Moreover, ASNS expression is positively associated with clinical response to PD-1-based neoadjuvant immunotherapy in NSCLC patients with lymph node metastases, a relationship that warrants further investigation through *in vivo* anti-PD-1 treatment studies. These findings were striking and highlighted ASNS as a potential biomarker affecting various stages of the CI cycle for predicting the effect of neoadjuvant anti-PD-1 therapy in NSCLC patients with LN metastasis, and also elucidated the underlying mechanism of immune remodeling caused by LN metastasis.

## Conclusion

In conclusion, we found that lung cancer cells with ASNS high expression were predisposed to metastasize in LNs. ASNS facilitated α-aminobutyric acid secretion to augment both the immunogenicity of lung cancer cells and the cytotoxicity of CD8+ T cells. And LN metastasis with high ASNS expression fostered the development of lymphocyte niches permissive for T cell activation, memory, as well as the production of TSL and TTSM in metastatic LNs, especially in the vicinity of metastatic foci, through mechanisms such as asparagine paracrine, thereby reshaping the immune microenvironment within both the primary tumor site and metastatic LNs, and improving the efficacy of neoadjuvant immunotherapy in NSCLC patients with LN metastasis.

## Supplementary Material

Supplementary methods, figures and tables.

## Figures and Tables

**Table 1:** Association Between Clinical Characteristics and MPR (n=18).

Characteristic	MPR(n=10)	NPR(n=8)	P value
Age, y			
Mean (SD)	58.6 (3.5)	51.4 (2.8)	0.0074
Sex			
Male	9	6	
Female	1	2	0.5588^b^
Histology			
Squamous	8	5	
Non-squamous	2	3	0.6078^b^
Smoking history			
Yes	8	6	
No	2	2	> 0.9999^b^
Clinical stage			
IIA	1	0	
IIB	1	2	
IIIA	6	6	
IIIB	2	0	0.3691^c^
Lymph node involvement at baseline			
Yes	10	8	
No	0	0	> 0.9999^b^
ASNS expression*			
Mean (SD)	83.6(39.6)	27.4 (31.4)	< 0.00001^a^

^a^p value is calculated by t test. ^b^p value is calculated using Fisher's exact test. ^c^p value is calculated using chi-squared test. *ASNS expression were detected by immune histochemistry.

## Abbreviations

Asn: Asparagine
ASNS: Asparagine Synthetase
LLC: Lewis Lung Cancer
LN: Lymph node
NSCLC: Non-small cell lung cancer
RT-PCR: Real-Time PCR
TdLN: Tumor-draining lymph node
TdLN-TTSM: TdLN-residing tumor-specific memory T cell
Tsl: Stem-like T cell
