# Supplementary material for: Lung Cancer Cell-intrinsic Asparagine Synthetase Potentiates Anti-Tumor Immunity via Modulating Immunogenicity and Facilitating Immune Remodeling in Metastatic Tumor-draining Lymph Nodes
Source: Int J Biol Sci. 2025 Oct 10;21(14):6501–21. doi: 10.7150/ijbs.114791 (PMC12594603; doi:10.7150/ijbs.114791)
Supplement: Supplementary file 1 — Supplementary methods, figures and tables. [file ijbsv21p6501s1.pdf]

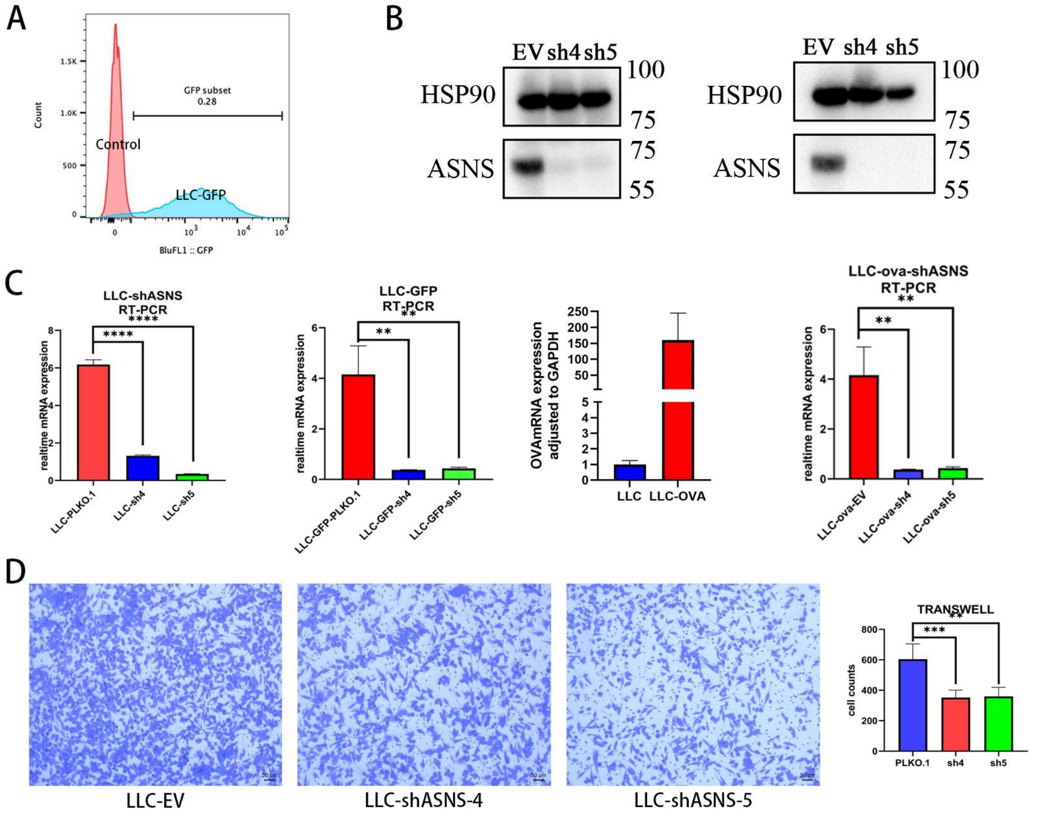

Supplemental Figure 1 ASNS enhanced cellular invasiveness of lung cancer cells. Figure 1A. LLC cells were labeled with Plenti-GFP-BSD (transfection of lentivirus), and FACS was used to verify the expression of GFP. **1B-C**. ASNS expression was knocked down in LLC, LLC-GFP and LLC-ova cells. The cells were infected with the shASNS lentivirus and control lentivirus (shNC). After the selection with puromycin, the resistant cells were pooled, and Western blotting(B) and RT-PCR(C) was used to detect the expression level of ASNS. **1D**. Invasion assay using the Transwell was conducted to quantify and analyse the effect of ASNS knockdown on the invasion of LLC cells.

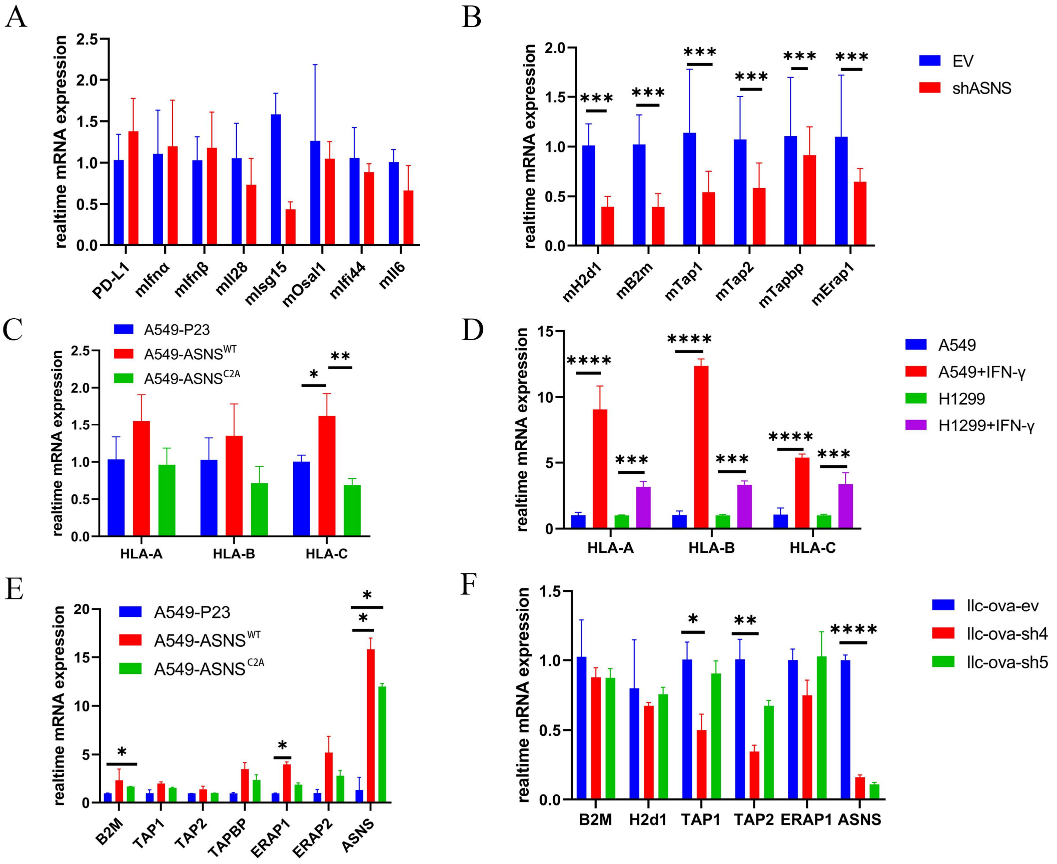

Supplemental Figure 2 ASNS was unable to regulate the mRNA level of type I IFN and interferon-stimulated genes (ISGs) or antigen presentation genes without treatment of IFN-γ. Figure 2A-B. RT-PCR analyses of relative mRNA levels of type I IFN and interferon-stimulated genes (ISGs) (A) or antigen presentation genes (B) in purified cancer cells derived from ASNS-knockdown (n=3) and control (n=3) LLC syngeneic tumors. **2C**. RT-PCR assays were used to detect the effects of $ASNS^{WT}$ and $ASNS^{C2A}$ over-expression on the induction of HLA mRNA level without treatment of IFN-γ. **2D**. RT-PCR assays were used to detect the effects of treatment of IFN-γ on the induction of HLA mRNA level. **2E**. RT-PCR assays were used to detect the effects of $ASNS^{WT}$ and $ASNS^{C2A}$ over-expression on the induction of the mRNA level of antigen presentation genes without treatment of IFN-γ. **2F**. RT-PCR assays were used to detect the effects of ASNS knockdown on the induction of the mRNA level of antigen presentation genes without treatment of IFN-γ.

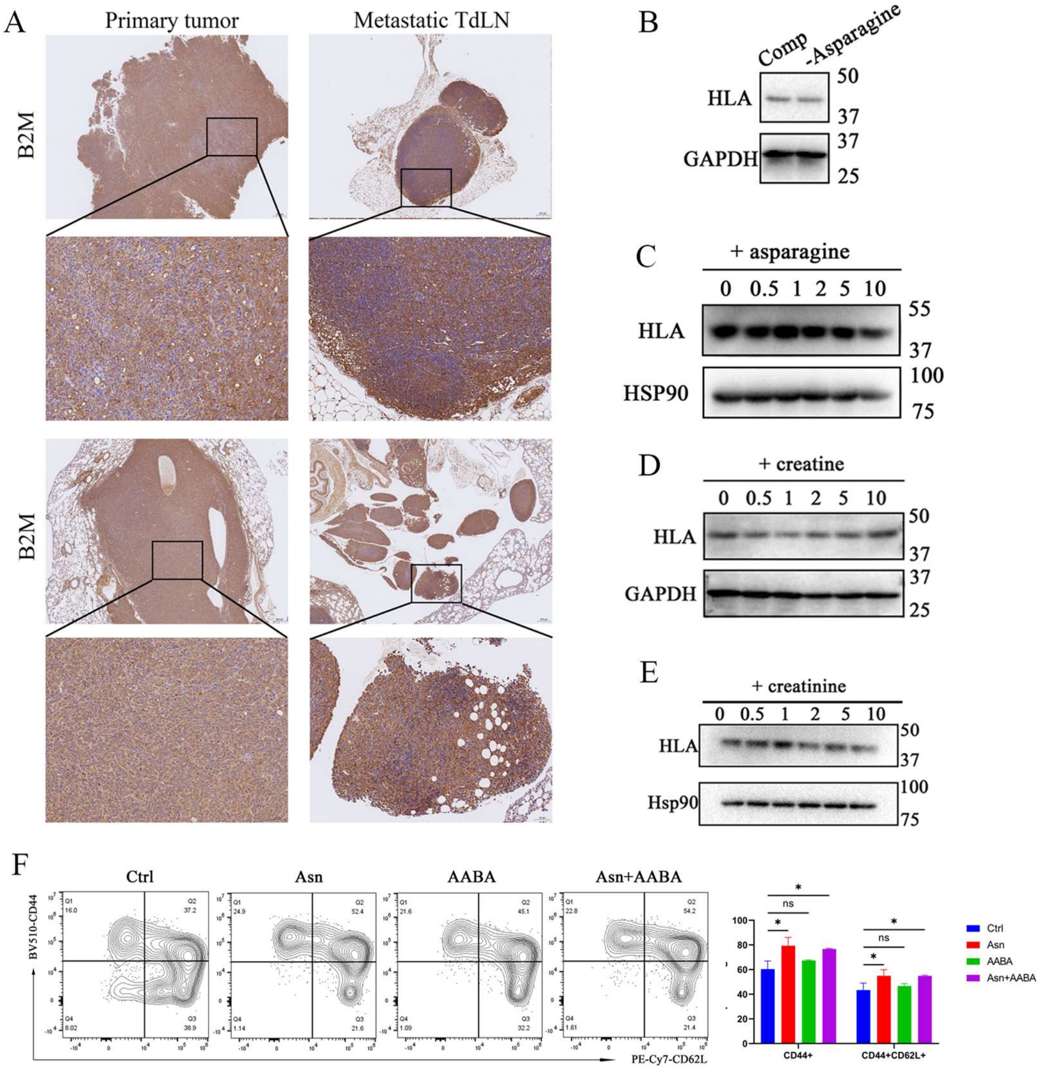

Supplemental Figure 3 ASNS enhances immunogenicity in lung cancer cells. Figure 3A. Representative IHC images showing the expression of B2M in primary tumor and LN metastasis from intrapulmonary implantation mouse model performed on C57BL/6 mice (upper, n=3) and popliteal lymph node implantation mouse model performed on C57BL/6 mice (below, n=4). **3B**. A549 cells were treated with low asparagine medium for 48 h. The expression of HLA was determined by western blotting. **3C-E**. A549 cells were treated with asparagine (C), creatine (D) or creatinine (E) for 48 h. The expression of HLA was determined by western blotting. **3F**. Representative FACS profiles are shown. OT-I CD8+T cells were treated with Asn, AABA or both for 48 h, and of the expression level of CD44 and CD62L was evaluated (n=3).

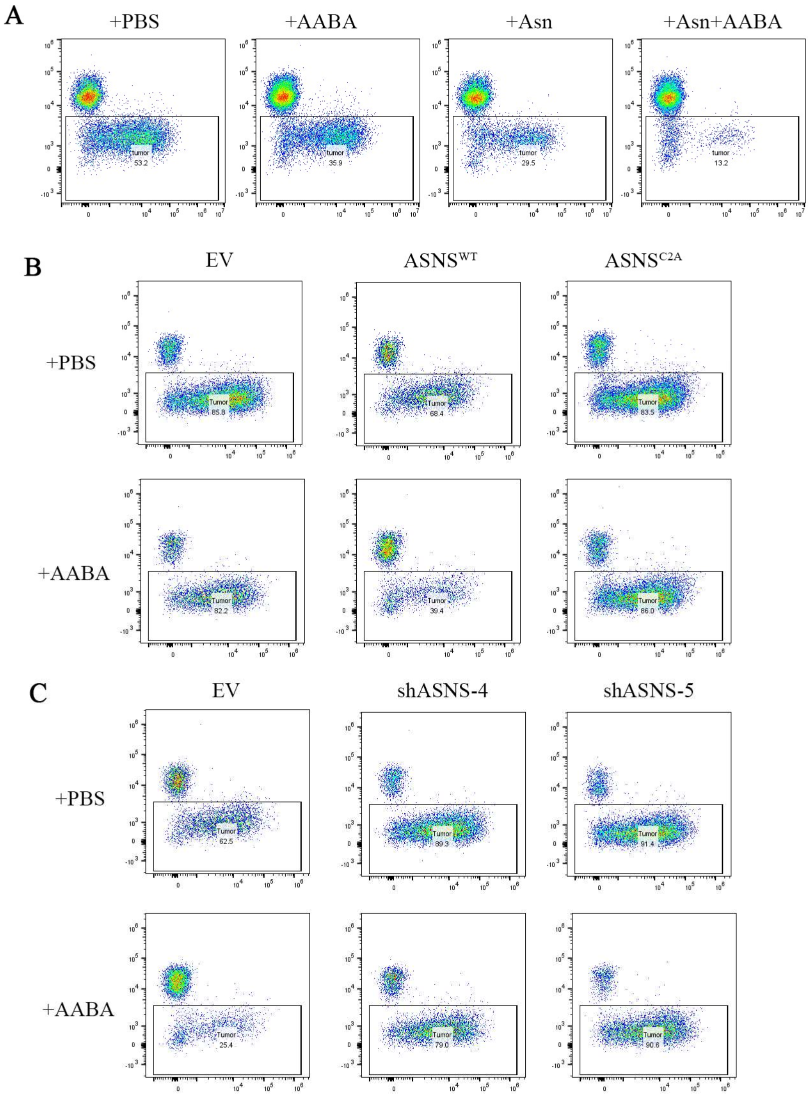

Supplemental Figure 4 ASNS promotes alpha-aminobutyric acid secretion to enhance the cytotoxicity of CD8+ T cells. **A.** Representative FACS profiles are shown. LLC-Ova cells were mixed with OT-I CD8+T cells at a 4:1 ratio and treated with Asn, AABA or both, and tumor cells viability was evaluated (n=3). **B.** Representative FACS profiles are shown. LLC-Ova cells with the overexpression of $ASNS^{WT}$ and $ASNS^{C2A}$ were mixed with OT-I CD8+T cells at a 4:1 ratio, and tumor cells viability was evaluated (n=3). **C.** Representative FACS profiles are shown. LLC-Ova cells with knockdown of ASNS were mixed with OT-I CD8+T cells at a 4:1 ratio, and tumor cells viability was evaluated (n=3).

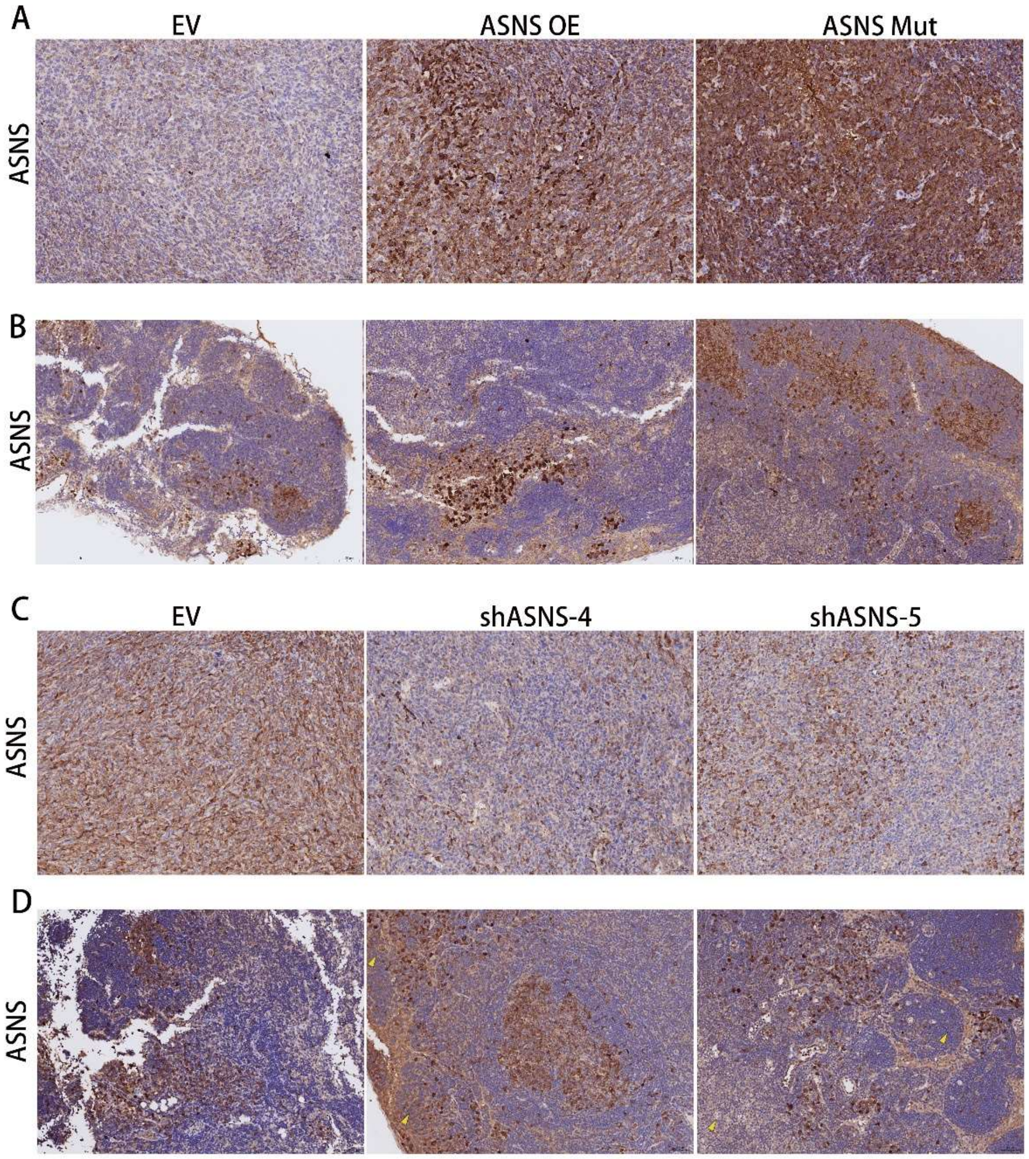

Supplemental Figure 5 LN metastasis model was conducted on C57BL/6 mice with LLC-ASNS overexpression cells. Figure4A-B. Representative IHC images showing the expression of ASNS in primary tumor(A) and LN metastasis(B) from footpad implantation mouse model performed on C57BL/6 mice (WT, n=5, MUT, n=4, and EV, n=3). **4C-D**. Representative IHC images showing the expression of ASNS in primary tumor(C) and LN metastasis(D) from footpad implantation mouse model performed on C57BL/6 mice (shASNS-4, n=3, shASNS-5, n=3, and EV, n=3).

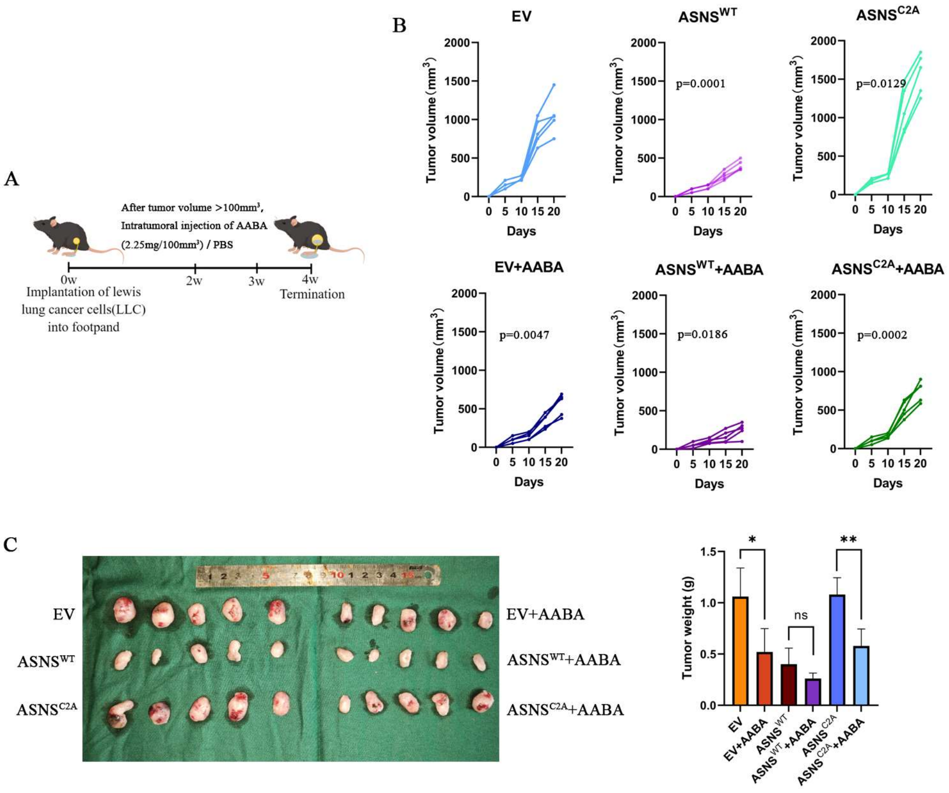

Supplemental Figure 6 Intra-tumoral AABA injection inhibits tumor progression. Figure5A. Schematics of intra-tumoral AABA injection performed on footpad implantation mouse model. **4B-D**. Tumor growth curve for footpad implantation mouse model receiving intra-tumoral AABA or vehicle (PBS) injections (2.25mg/100mm^3) every day after tumor volume ⩾100mm^3(WT, n=5, MUT, n=5, and EV, n=5), and final tumor weight(C-D) was measured and analyzed.

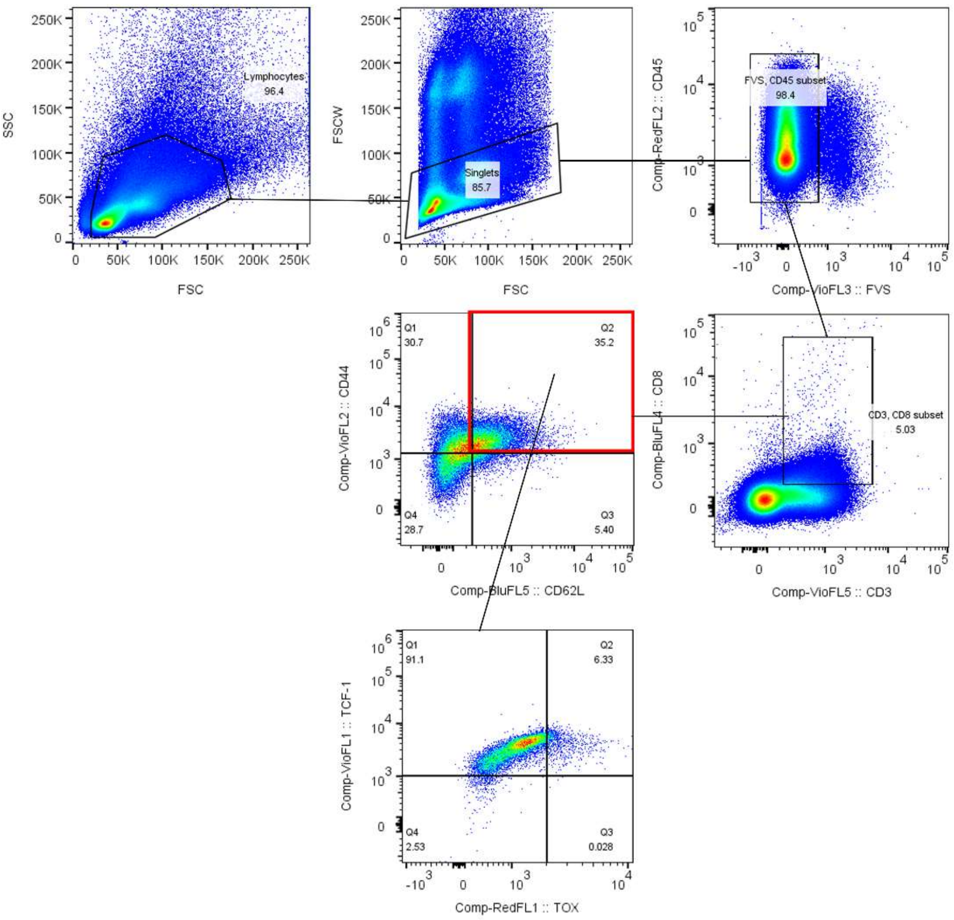

Supplemental Figure 7 Gating strategy used to identify TTSM subsets in TdLN.

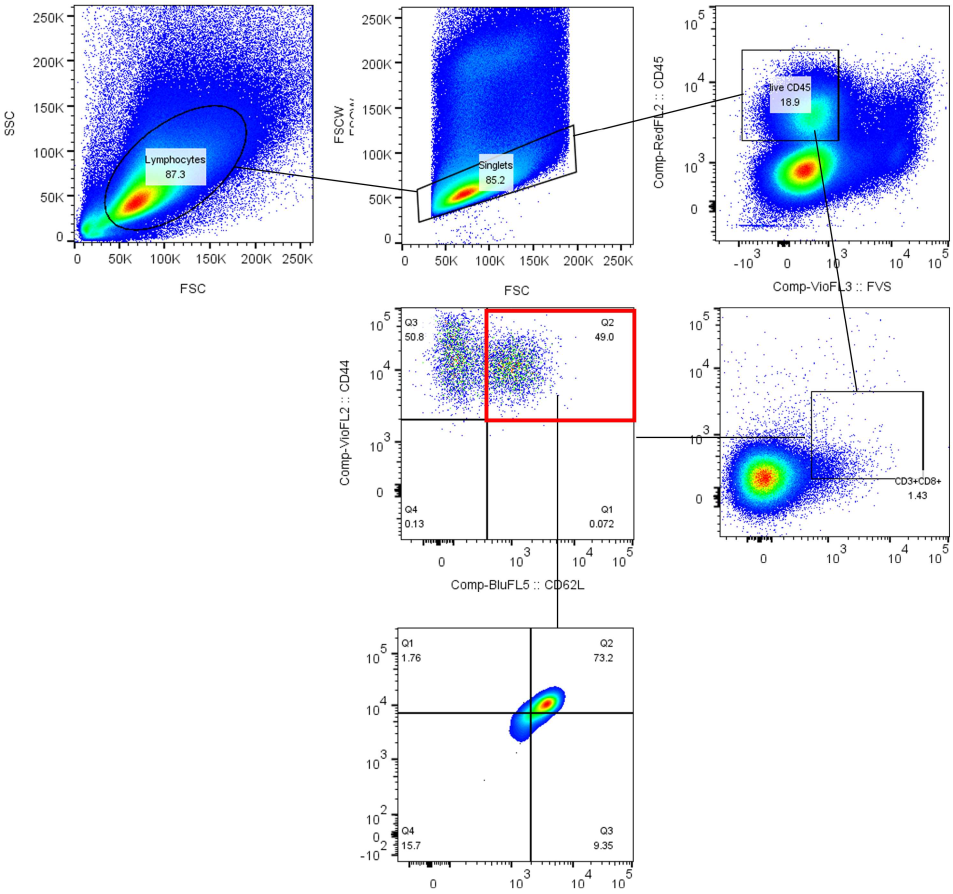

Supplemental Figure 8 Gating strategy used to identify TTSM subsets in primary tumor.

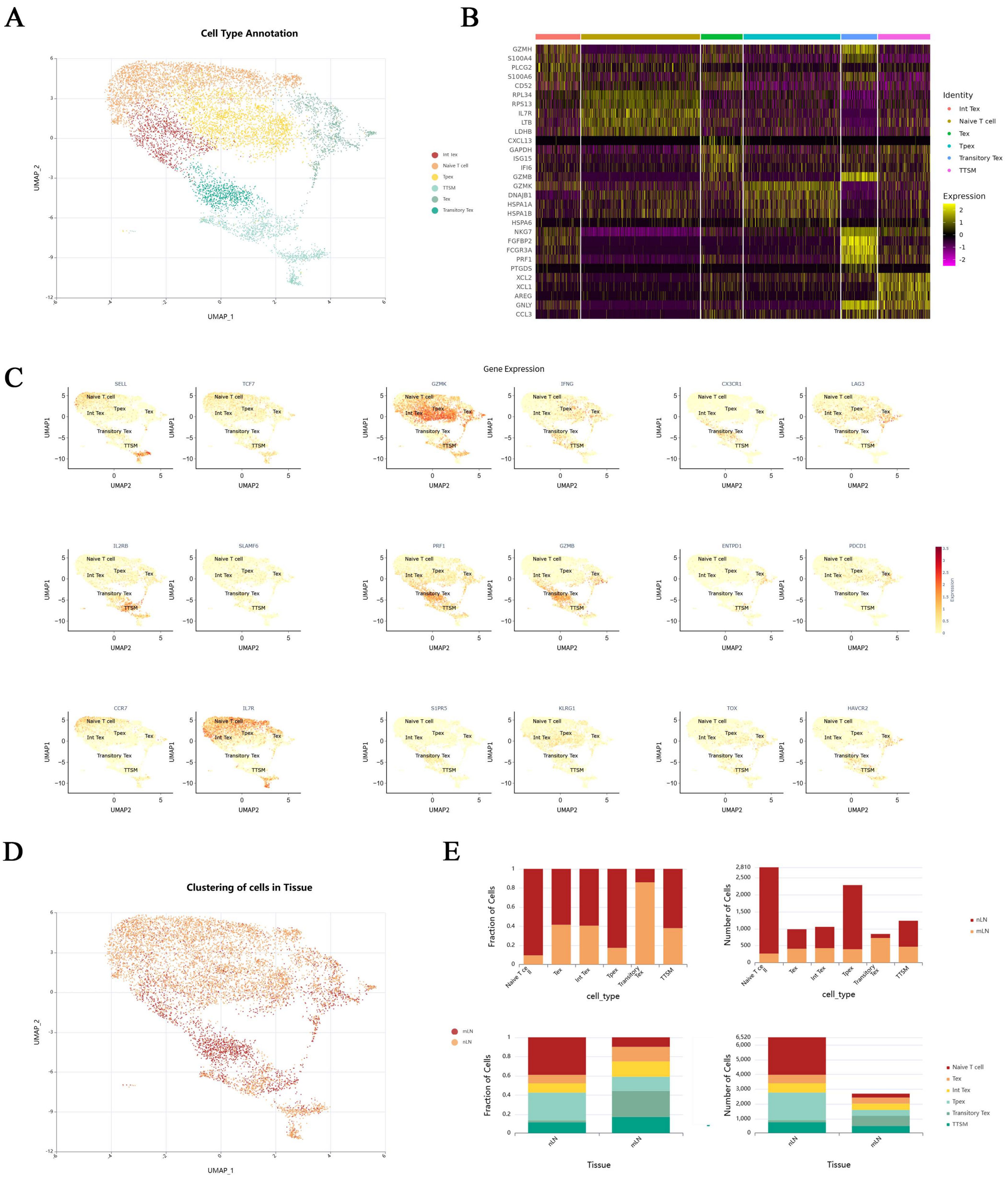

Supplemental Figure 9 Transcriptional profiling of CD8+ T cells derived from metastatic or non-metastatic TdLN by scRNA-seq. **Figure9A**. Uniform manifold approximation and projection (UMAP) visualization depicts scRNA-seq clusters of CD8^{+} T cells (n = 9,216) from 16 samples. **9B**. Heatmap illustrating differentially expressed (DE) genes across clusters, ranked by fold change. **9C**. Single-cell transcription levels of representative genes, with expression levels color-coded (yellow: not expressed; red: expressed). **9D-E**. UMAP visualization (D) and bar plot (E) displaying the proportion and absolute number of cells per group within each cluster, as well as the distribution of clusters within each group.

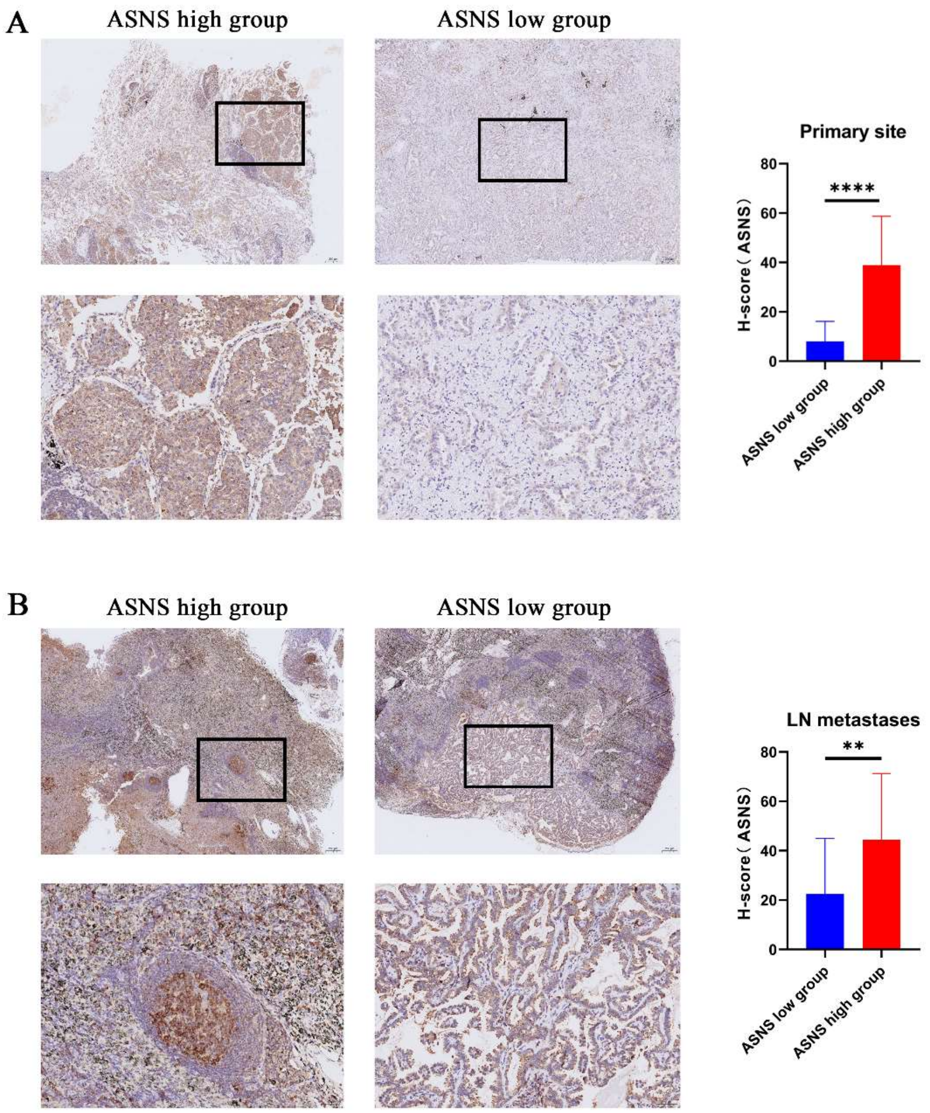

Supplemental Figure 10 Discordance of ASNS expression was observed in NSCLC patients. Figure8A-B. Representative IHC images showing the expression of ASNS in primary tumor(A) and LN metastasis(B) from NSCLC patients (ASNS high group, n=7, and ASNS low group, n=6).

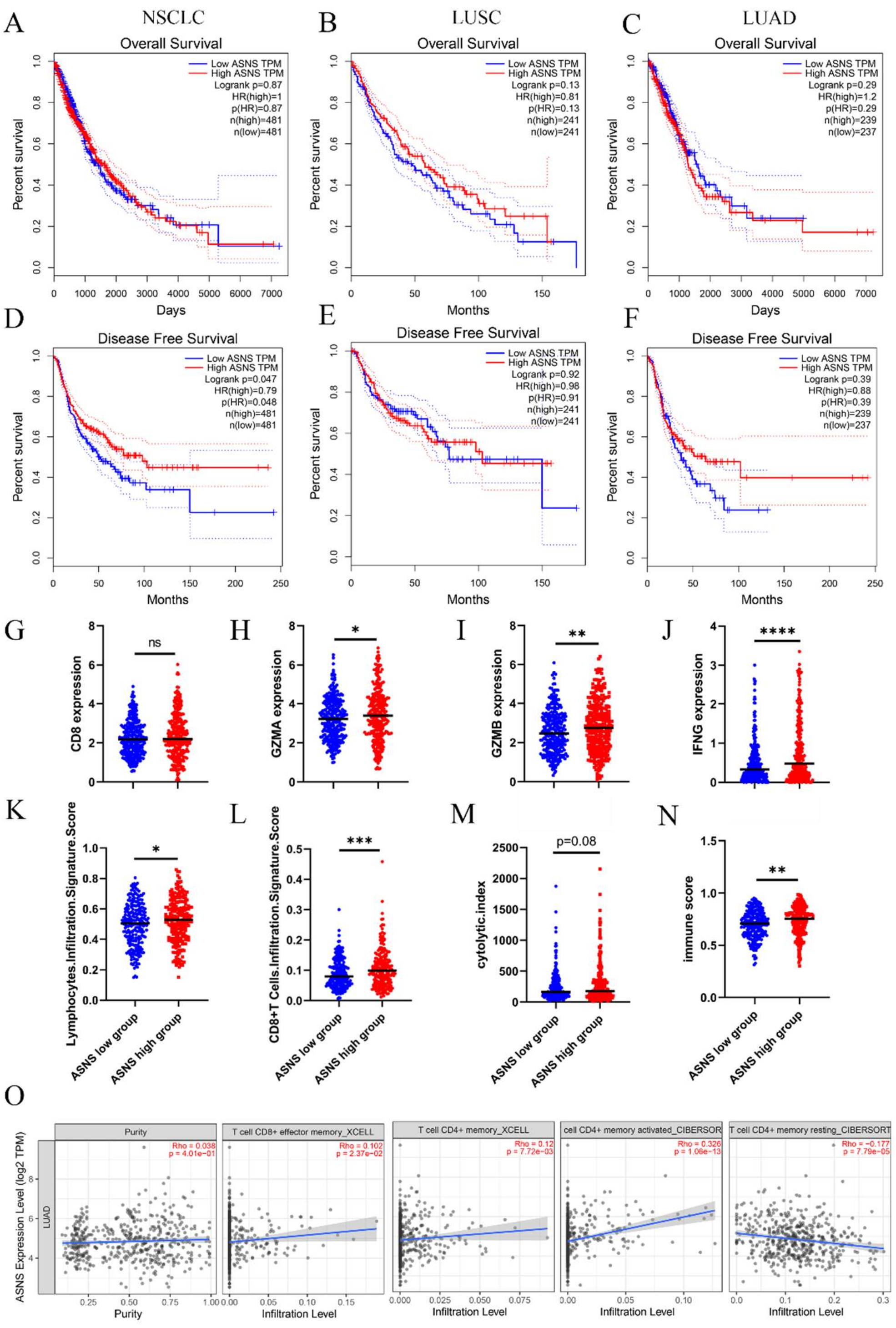

Supplemental Figure 11 Survival analysis and immune infiltration analysis of ASNS in NSCLC. 9A-C. Overall survival analysis of ASNS in NSCLC(A), LUSC(B) and LUAD(C) based on TCGA dataset. **9D-F**. Disease free survival analysis of ASNS in NSCLC(D), LUSC(E) and LUAD(F) based on TCGA dataset. **9G-J**. Correlation between ASNS expression and CD8(G), GZMA(H), GZMB(I) and IFNG(J) expression based on TCGA dataset. **9K-N**. Correlation between ASNS expression and lymphocytes infiltration signature score (K), CD8+T Cells infiltration signature score (L), cytolytic index (M) and immune score (N) based on TCGA dataset.

9O. Correlation between ASNS expression and TIL infiltration, memory and activation in NSCLC based on Timer2.0 dataset.

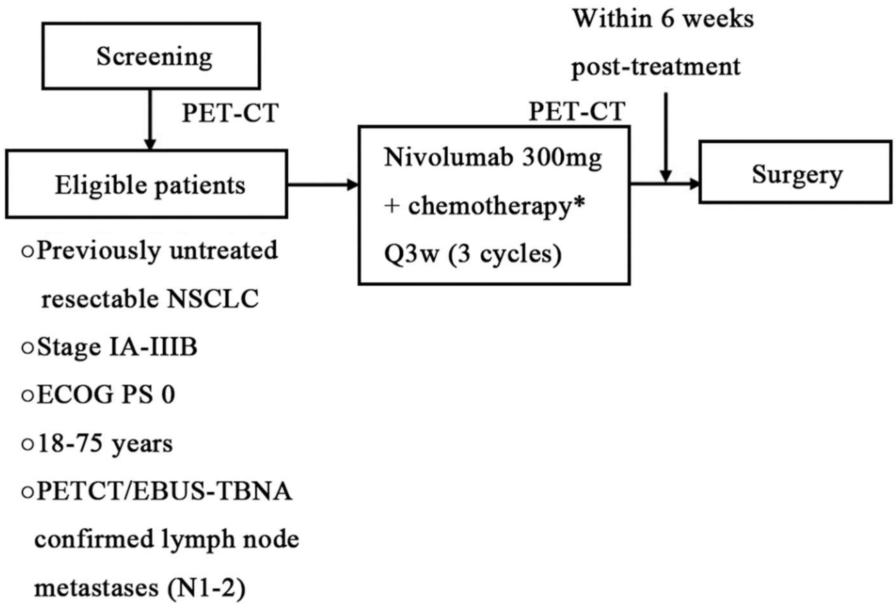

Supplemental Figure 12 Study design.

* Pemetrexed + cisplatin (nonsquamous only), or paclitaxel + carboplatin.

Supplemental Table1. ShRNA primers.

Name	Sequence
shASNS-m-1F	CCGGCGTGAAGAACAATCTGCGTATCTCGAGATACGCAGATTGTTC TTCACGTTTTTG
shASNS-m-1R	AATTCAAAAACGTGAAGAACAATCTGCGTATCTCGAGATACGCAG ATTGTTCTTCACG
shASNS-m-2F	CCGGGCCAGATATGAGAATTCCAAACTCGAGTTTGGAATTCTCATA TCTGGCTTTTTG
shASNS-m-2R	AATTCAAAAAGCCAGATATGAGAATTCCAAACTCGAGTTTGGAATT CTCATATCTGGC
shASNS-m-3F	CCGGCCCTTATTTGTGGCTCTGTTACTCGAGTAACAGAGCCACAAA TAAGGGTTTTTG
shASNS-m-3R	AATTCAAAAACCCTTATTTGTGGCTCTGTTACTCGAGTAACAGAGC CACAAATAAGGG
shASNS-m-4F	CCGGCCCAGAAGTTTCCCTTCAATACTCGAGTATTGAAGGGAAACT TCTGGGTTTTTG
shASNS-m-4R	AATTCAAAAACCCAGAAGTTTCCCTTCAATACTCGAGTATTGAAGG GAAACTTCTGGG
shASNS-m-5F	CCGGCGCTATCAAGAAACGCTTGATCTCGAGATCAAGCGTTTCTTG ATAGCGTTTTTG
shASNS-m-5R	AATTCAAAAACGCTATCAAGAAACGCTTGATCTCGAGATCAAGCGT TTCTTGATAGCG
shASNS-h-1F	CCGGGCTGTATGTTCAGAAGCTAAACTCGAGTTTAGCTTCTGAACA TACAGCTTTTTG
shASNS-h-1R	AATTCAAAAAGCTGTATGTTCAGAAGCTAAACTCGAGTTTAGCTTC TGAACATACAGC
shASNS-h-2F	CCGGCGAGTGAAGAAATATCCGTATCTCGAGATACGGATATTTCTTC ACTCGTTTTTG
shASNS-h-2R	AATTCAAAAACGAGTGAAGAAATATCCGTATCTCGAGATACGGATA TTTCTTCACTCG

Supplemental Table2. PCR primers

Gene	Forward	Reverse
GAPDH	AGGTCGGTGTGAACGGATTTG	TGTAGACCATGTAGTTGAGGTCA
ASNS	GTTGACCCGCTGTTTGGAAT	TGGTTTTCTCGATGCCTCCT
hB2M	GAGGCTATCCAGCGTACTCCA	CGGCAGGCATACTCATCTTTT
hHLA-A	ACCCTCGTCCTGCTACTCTC	CTGTCTCCTCGTCCCAATACT
hHLA-B	CAGTTCGTGAGGTTCGACAG	CAGCCGTACATGCTCTGGA
hHLA-C	GGACAAGAGCAGAGATACACG	CAAGGACAGCTAGGACAACC
hTAP1	CTGGGGAAGTCACCCTACC	CAGAGGCTCCCGAGTTTGTG
hTAP2	TGGACGCGGCTTTACTGTG	GCAGCCCTCTTAGCTTTAGCA
hTAPBP	CCTGGAGGTAGCAGGTCTTTC	ATCCTTGCAGGTGGACAGGTA
hERAP1	CCCCTCAAATGGTCCCTTGC	GAGATGCTTCAGTGCTCTGAC
hERAP2	CACTAATGGGGAACGATTTCCTT	CTGACCAAGACTTCGATCTTCTC
mH2d1	GTACCTGCAGTTCGCCTATGA	TAATGCTCTGCAGCACCACTCT
mB2m	TTCTGGTGCTTGTCTCACTGA	CAGTATGTTCGGCTTCCCATTC
mTap1	GGACTTGCCTTGTTCCGAGAG	GCTGCCACATAACTGATAGCGA
mTap2	CTGGCGGACATGGCTTTACTT	CTCCCACTTTTAGCAGTCCCC
mTapbp	GGCCTGTCTAAGAAACCTGCC	CCACCTTGAAGTATAGCTTTGGG
mErap1	TAATGGAGACTCATTCCCTTGGA	AAAGTCAGAGTGCTGAGGTTTG
